# Findings from a three-round Delphi study: essential topics for interprofessional training on complementary and integrative medicine

**DOI:** 10.1186/s12906-020-03140-x

**Published:** 2020-11-17

**Authors:** Angelika Homberg, Nadja Klafke, Svetla Loukanova, Katharina Glassen

**Affiliations:** Department of General Practice and Health Services Research, University Hospital Heidelberg, Im Neuenheimer Feld 130.3, 69120 Heidelberg, Germany

**Keywords:** Medical education, Interprofessional education, Complementary medicine, Integrative medicine, Interprofessional collaboration, Curriculum development, Delphi study

## Abstract

**Background:**

Integrating complementary medicine into medical care promotes patient-oriented care. A well-informed and collaborative professional healthcare team is essential for effective and patient-safe implementation of these methods. At present, the skills for patient counseling, therapy and care regarding complementary medicine vary among the professional groups involved. Professionals generally feel that they are not sufficiently qualified in this area. Curricular concepts for Complementary and Integrative Medicine (CIM) are virtually non-existent in undergraduate interprofessional training. The aim of this study is to initiate a consensus-building process between various experts (professionals, students, patient and faculty representatives) in order to identify which topics should be the focus of such a curriculum.

**Methods:**

A three-round Delphi study was carried out from March 2018 to March 2019 to compile the experience and knowledge of experts in the field of integrative patient care and interprofessional education. Sixty-five experts from Germany and German-speaking Switzerland with various professional backgrounds and experiences were asked to name general content, therapy methods and treatment reasons which should be addressed in interprofessional seminars. In the subsequent rounds these were rated on a seven-point Likert scale. The ratings were assigned to relevance groups and discussed in a final workshop in July 2019.

**Results:**

The response rates for the three rounds were 76% (*n* = 50), 80% (*n* = 40) 90% (*n* = 36); and 21% (*n* = 11) for the final workshop. The experts suggested that topics could be aligned along the most common treatment reasons such as insomnia, generalized pain, fatigue and back pain. However, it is important that students also receive an overview of the evidence base for different therapeutic concepts, especially in the field of classical natural medicine, acupuncture and mind-body medicine, and that they get an overview of the effects and interactions of frequently used procedures.

**Conclusion:**

Consensus was reached among the various experts on the most important topics for an interprofessional CIM curriculum. The systematic evaluation of the topics in this study can help to create a curriculum that achieves a high level of acceptance among teachers, lecturers and students, and thus facilitates implementation at universities and medical faculties.

**Supplementary Information:**

The online version contains supplementary material available at 10.1186/s12906-020-03140-x.

## Background

Complementary medicine is an important and often underestimated part of health services, which has long been used to maintain health and to prevent and treat diseases, especially chronic diseases [1]. In Europe and North America, complementary medicine is often used for health-related problems such as pain, allergies and oncological diseases, in addition to biomedical therapy and care [2], and is also increasingly included in clinical guidelines [3–9]. The request for additional therapy options often emanates from the patients and is sometimes applied on their own initiative, without consulting professional providers or even disclosing the application to them [10]. This entails considerable risks for the patients because complementary procedures also have side-effects and interactions with conventional treatment. In addition, the range of complementary and alternative options is very broad and patients often cannot distinguish between reliable and non-reliable therapies [11]. To implement complementary medicine into patient care, it is important that all of the professionals involved are well-informed and have a uniform basic knowledge of complementary medicine. The majority of the health professionals perceive their knowledge as low and they do not feel able to advise patients appropriately [12–15]. They also express their interest in integrating complementary medicine content into their undergraduate studies [16, 17] and the need to learn more about the roles of other health professionals in providing CIM [18, 19].

It is nearly impossible for an individual practitioner to have an overview of the entire field of non-conventional approaches and to offer the best possible treatment options for the individual patient over the entire course of a disease. Therefore, both conventional and non-conventional methods must be included in the decision-making process. *Integrative medicine* itself means both the integration of conventional and complementary procedures and the integration of all professions involved in the provision of care: “Integrative medicine reaffirms the importance of the relationship between practitioner and patient, focuses on the whole person, is informed by evidence, and makes use of all appropriate therapeutic and lifestyle approaches, healthcare professionals and disciplines to achieve optimal health and healing” [20].

Patients bring up their concerns with different professional groups. To ensure patient safety and build trust, it is important that professionals are well-coordinated among themselves. Being educated in an interprofessional setting provides an opportunity to share skills and knowledge between the professionals and facilitates the development of shared values as well as a better understanding of the roles and responsibilities of the other healthcare professionals [21]. According to the definition from the Centre for the Advancement of Interprofessional Education (CAIPE, 2002) “Interprofessional learning takes place when members or students of two or more professions learn with, from and about each other to improve collaboration and the quality of care and services” [22].

In order to meet the demand for holistic care and foster future collaboration in professional practice, it seems beneficial to teach complementary and integrative medicine (CIM) in an interprofessional setting [23, 24] as this can promote patient-centered and team-based care and save resources in education [18, 19, 25]. In addition, interprofessional teaching on CIM can also broaden the range of options for individual caregivers and providers, improve outcomes for people with chronic diseases and lead to cost savings in the healthcare system [26].

One difficulty for medical schools, however, is selecting topics in such a way that they are relevant for all of the professional groups attending interprofessional training programs. In the field of complementary medicine, experience plays a major role, as there is sometimes little evidence available for the procedures or corresponding proof is still missing. The various complementary and natural medical procedures play a different role in the individual professional groups; for example, there are specific training opportunities, such as acupuncture for doctors, hydrotherapy for physiotherapists, and packings and aromatherapy for nurses [27, 28]. When selecting suitable topics for interprofessional training programs, different interest groups come into contact with each other, and this can hinder the development or implementation. The aim of this study was to use a Delphi survey in a multi-stage, structured survey process to reach a consensus among experts on relevant topics for such a curriculum.

## Methods

A three-round Delphi study was conducted to identify which topics are relevant for an interprofessional CIM curriculum for medical and other healthcare students, like nurses, physiotherapists and midwives. The Delphi method is particularly suitable for achieving equal participation of experts at different hierarchical levels and avoiding social pressure and distortions [29].

Since the results of a Delphi survey are largely determined by the composition of the expert group, particular care was taken in its selection [30]. The following selection criteria were defined by a steering group (AH, KG, NK, SL, patient representative, teaching coordinator and course administrator):

- The number of participants from medical and other health professions (nurses, physiotherapists and midwives) should be represented in roughly equal parts.
- Doctors are to be recruited from the outpatient and inpatient sector and from different specialist areas, whereby pediatrics and geriatrics should definitely be represented.
- In order to achieve general acceptance of the developed curriculum, experts with different complementary medical backgrounds should be invited, as well as those who do not practice CIM or who are critical towards it.
- Patient representatives, insurance representatives and some stakeholders, for instance institute directors and an academic dean, should also be invited to enrich the Delphi process with the perspectives of patients and medical faculties.
- Medical and healthcare students should be involved in order to cover the learners’ perspectives.

As a relatively high drop-out rate can be expected in long-term and multi-round procedures, we have tried to recruit several persons for each area. The actual selection of the experts took place throughout Germany and German-speaking Switzerland. Individuals who met the criteria were proposed by the steering group, others were recruited through professional associations, for example membership of the Society for Medical Education or the Association of Representatives of German Students of Medicine and Physiotherapy. It was expected that dedicated experts would be more motivated and willing to participate. As a result, sixty-five experts from Germany and German-speaking Switzerland were invited to participate in the consensus process from March 2018 to March 2019.

The data was collected with online-questionnaires in each round (supplementary file). A pre-test with members of the steering group and three external persons was conducted in advance for each round. The questionnaire was checked for comprehensibility, content and technical feasibility.

The survey was strictly anonymous; however, the main researcher (AH) knew the participants’ personal data in order to control the survey process.

The participants were personally invited by e-mail. A reminder was sent for each round after 3 weeks and after 6 weeks. Participation in the previous round was required to participate in the following round. Non-participants were excluded from further involvement in the Delphi rounds. After each round the participants received a comprehensive result report in which all results were presented.

The online questionnaires for the three Delphi rounds comprised several parts. Each of these questionnaires contained a set of questions on content issues. In the first Delphi round experts were asked to rate ten general topics and 15 complementary therapy methods on a seven-point Likert scale (1 = not at all relevant, 7 = very relevant). These items were compiled based on existing CIM curricula for undergraduate academic medical and other healthcare students [31–34]. All of the given general topics and therapy methods/concepts are shown in Tables 2 and 3. It was specified that the experts should rate the topics by envisioning a teaching program for approximately 20 undergraduate medical and other healthcare students, and with 15 lessons of 45 min each. The experts were able to indicate for each topic whether they felt competent to assess it. Assessments of experts who did not feel competent were not considered in the respective consensus calculations [30]. According to the experts’ assessments, the topics and therapy methods were assigned to four relevance groups. Group 1: highly relevant (at least 80% of the respondents rated the topics with a 6 or 7 on the Likert scale), Group 2: relevant (at least 80% rated the remaining topics with at least a 5), Group 3: partially relevant (at least 80% rated the remaining topics with at least a 4), and Group 4: not relevant (for the remaining topics). Furthermore, experts were asked to suggest additional topics and methods in free-text fields. The topics proposed by the experts in the first and second rounds were categorized using content analysis and presented for evaluation in the respective following round.

The experts were also given the opportunity to add further remarks. Since the follow-up rounds were developed on the basis of the remarks from the previous rounds, the subsequent survey questionnaires were designed to reflect the experts’ opinions. At the end of the third round, experts were asked whether they were willing to participate in a day-long final consensus workshop.

The results from all Delphi rounds were consolidated at this final workshop in July 2019 to which all participants involved in the previous Delphi study were invited. Due to the distribution of the experts throughout Germany and Switzerland and their involvement in everyday working life, it was to be expected that the number of participants would be small. According to Turtuff, a small group of people is sufficient to bundle the results and discuss possible applications: “Once the Delphi has been accomplished, a small workable committee can utilize the results to formulate the required policy” ([35] p.83). The aim of the workshop was to discuss the topics classified as relevant and very relevant and the advantages and disadvantages of structuring the content on the basis of general topics, therapy methods, treatment reasons or concrete patient cases.

## Results

The response rates for the three rounds were 76% (*n* = 50), 80% (*n* = 40) and 90% (*n* = 36). In the third Delphi round, 15 participants agreed to participate in the final workshop. A date was set with the willing participants by means of a data query. Only six participants gave a binding commitment, so all participants who took part in at least one Delphi round and the three external pre-testers were also invited. By doing this, it was possible to receive 12 binding commitments; one participant cancelled at short notice, leaving eleven experts to take part in the final workshop (11 out of 52, response rate 21%). The characteristics of the experts are shown in Table 1.

**Table 1:** Participant characteristics in the three Delphi rounds and final workshop

		Round 1	Round 2	Round 3	Workshop
		*n* = 50 (%)	*n* = 40 (%)	*n* = 36 (%)	*n* = 11 (%)
Gender	female	36 (72)	31 (78)	27 (75)	11 (100)
	male	14 (28)	9 (23)	9 (25)	0
Country	German	47 (94)	37 (93)	33 (92)	11 (100)
	German-speaking Switzerland	3 (6)	3 (8)	3 (8)	0
Age	mean (SD) (range)	48 (11.9) (24–66)	48 (11.7) (24–66)	48 (11.8) (24–66)	47 (9.6) (26–58)
Professional field^a^	Medicine	29 (58)	21 (53)	19 (53)	3 (27)
	Therapy	10 (20)	10 (25)	9 (25)	0
	Nursing	10 (20)	9 (23)	8 (22)	3 (27)
	Midwifery	3 (6)	3 (8)	3 (8)	1 (9)
	Social work/Public health	4 (8)	4 (10)	3 (8)	0
	Other science	5 (10)	4 (10)	4 (11)	2 (18)
	Patient representative	3 (6)	2 (5)	1 (3)	0
	Student	5 (10)	3 (8)	3 (8)	2 (18)
Experience in education (educ.)^a^	Academic educ. (≥ 5 years)	30 (60)	25 (63)	22 (61)	–
	Interprofessional educ.	24 (48)	20 (50)	18 (50)	–
	Faculty development	22 (44)	18 (45)	16 (44)	–
	Curriculum development	28 (56)	25 (63)	22 (61)	–
Experience in patient care ≥ 3 years^a^	Inpatient care	28 (56)	23 (58)	21 (58)	–
	Outpatient care	27 (54)	23 (58)	21 (58)	–
	Prevention	19 (38)	18 (45)	15 (42)	–
	Palliative care	6 (12)	4 (10)	4 (11)	–
	Rehabilitation	15 (30)	11 (28)	10 (28)	–
	Patient counseling	25 (50)	20 (50)	18 (50)	–
Application of CIM methods in professional practice^b^	Phytotherapy	17 (34)	14 (35)	14 (39)	17 (34)
	TCM, Acupuncture	10 (20)	9 (23)	9 (25)	10 (20)
	Mind-body medicine	9 (18)	8 (20)	7 (19)	9 (18)
	Hydrotherapy	8 (16)	6 (15)	6 (17)	8 (16)
	Homeopathy	6 (12)	5 (13)	5 (14)	6 (12)
	Exercise therapy	6 (12)	5 (13)	4 (11)	6 (12)
	Aromatherapy	5 (10)	3 (8)	3 (8)	5 (10)
	Anthroposophical medicine	4 (8)	3 (8)	3 (8)	4 (8)
	Dietetics	3 (6)	3 (8)	2 (6)	3 (6)
	Manual medicine	3 (6)	2 (5)	2 (6)	3 (6)
	Massage	3 (6)	2 (5)	2 (6)	3 (6)
	Acupressure	2 (4)	1 (3)	1 (3)	2 (4)
	Other methods^c^	7 (14)	4 (10)	3 (8)	7 (14)
	No CIM application	24 (28)	21 (53)	17 (47)	24 (28)

*Note.* Data are based on self-disclosure of the participants in the first round of the survey. ^a^Multiple choice question, multiple answers possible; ^b^Text boxes, multiple answers possible; − = not recorded; ^c^Bach flowers, light-, breath-, autohaemo-, orthomolecular-, neural therapy, diverting procedures

**Fig. 1:**
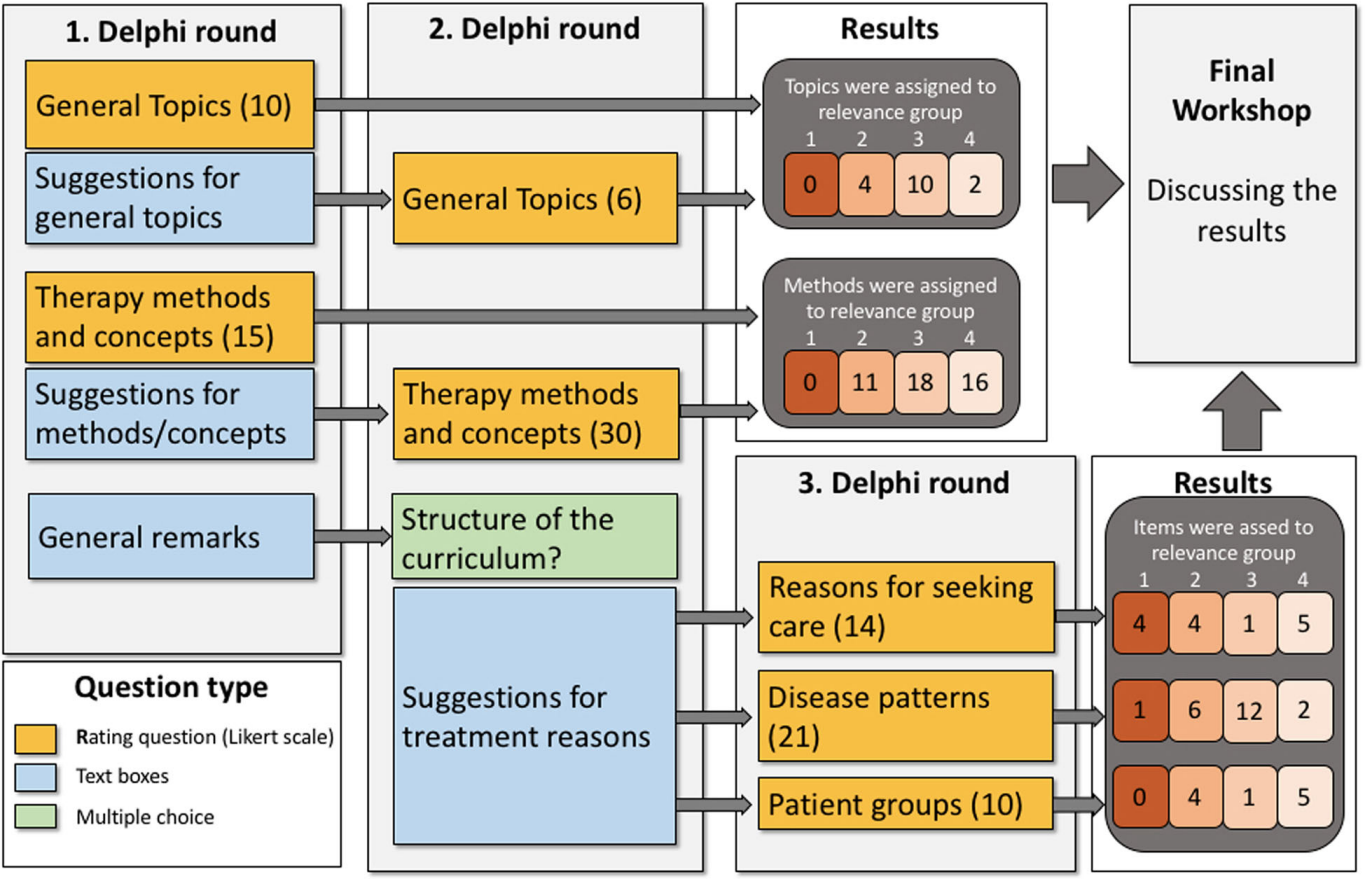
Overview of the three rounds of the survey process, workshop and results*.* First Delphi round: *n* = 50; second Delphi-round: *n* = 40; third Delphi-round: *n* = 36; final workshop *n* = 11

A total of 16 general topics and 45 therapy methods were assessed in rounds 1 and 2. In the third round, 45 proposals were made for treatment reasons. The survey process and the number of points assigned to the respective relevance groups are shown in Fig. 1.

### General topics

Ten general topics were given in the first Delphi round. The experts could list other suggestions in free-text fields. This option was used by 12 experts; however, some suggestions concerned didactic implications or methodological therapeutic aspects and were considered in the evaluation of these areas. From these suggestions, six other general topics were identified, which were then submitted for evaluation in the second round. For the analysis, all 16 general topics (first round: 10; second round: 6) were assigned to relevance groups. No topic could be assigned to relevance group 1; the results are shown in Table 2.

**Table 2:** Relevance groups for the general topics on CIM

Delphi round	Rating Question^a^ ***Please assess the topics in terms of their relevance for an interprofessional teaching program.***	Consensus^b^
**Group 2: Relevant**^c^		
1	Overview of methods in classical natural medicine	94
1	Clarification and explanation of CIM terms	88
1	Overview of methods in non-classical natural medicine	88
1	Overview of effects and interactions of selected CIM therapies	84
**Group 3: Partially relevant**^d^		
1	Legal issues relating to CIM therapies	92
1	Deepening the assessment of CIM evidence on the basis of selected studies	92
2	Placebo	92
2	Motivation for the demand for CIM services in the population	89
1	Use of CIM therapies	89
2	CIM in guidelines: Information and critical reflection	89
2	Motives and reasons for using CIM therapies	87
1	Supply structures and service providers	86
2	Health of health professionals, resilience and burn-out prevention	81
1	Overview of CIM databases and literature search via the Internet	80
**Group 4: Not relevant**^e^		
2	Obstacles to and opportunities for using CIM from a provider perspective	78
1	Historical and philosophical backgrounds of CIM therapy	77

*Note.* Results from the first (n = 50) and second (n = 40) Delphi-rounds. ^a^Seven-point Likert scale (7 = very relevant, 1 = not at all relevant); ^b^Percentage of ratings on Likert scale 5–7 in Group 2, and Likert scale 4–7 in Group 3 and 4; ^c^Topics are assigned to Group 2 if at least  80% of the respondents gave a rating of 5–7 on the Likert scale; ^d^Topics are assigned to group 3 if at least 80% of respondents gave a rating of 4–7 on the Likert scale; ^5e^Topics are assigned to group 4 if < 80% of respondents gave a rating of 4–7 on the Likert scale

### Therapy methods and concepts

CIM therapy methods and concepts were also collected and justified in the first two rounds following the same procedure as for the general content. Fifteen therapy methods and concepts were specified in the first round. From the free-text fields for the first round, 30 additional therapy methods were identified, which were then submitted for evaluation in the second round. The results (in total 45 therapy methods and concepts) are shown in Table 3. As with the general topics, no specific topics could be assigned to relevance Group 1.

**Table 3:** Relevance groups for the CIM therapy methods

Delphi round	Rating Question^a^ ***Please assess the topics in terms of their relevance for an interprofessional teaching program.***	Consensus^b^
**Group 2: Relevant**^c^		
1	Overview: Movement therapy	91
2	General nutritional recommendations	89
1	Overview: Phytotherapy	87
1	Overview: Regulative therapy / Mind-Body medicine	87
1	Overview: Dietetics	85
2	Progressive muscle relaxation	83
2	Mindfulness and meditation	82
2	Exercise and endurance training	82
1	Overview: Hydrotherapy	81
2	Nutrition for food intolerances and allergies	81
2	Stress management	80
**Group 3: Partially relevant**^d^		
1	Acupuncture	89
1	Yoga	89
2	Chiropractic	87
2	Tai Chi	87
1	Traditional Chinese Medicine (TCM)	86
2	Dance and music therapy	86
2	Diet for special wishes (Mediterranean, vegan, low-carb, etc.)	86
2	Aromatherapy, embrocation	85
2	Wraps and compresses	85
2	Kneipp therapy	85
2	Classical massages	85
2	Art therapy	84
2	Digital forms of relaxation	84
2	Fascia therapy	84
2	Medicinal plants, tea, tinctures	83
2	Shiatsu	83
2	Osteopathy	82
2	Fasting	80
**Group 4: Not relevant**^e^		
1	Neural therapy	79
1	Homeopathy	76
1	Anthroposophical medicine	74
2	High-dose vitamins (orthomolecular therapy)	73
2	(Foot zone) reflex therapy	71
1	Cranio-sacral therapy	70
1	Diverting procedures	68
1	Ayurveda	67
1	Tibetan medicine	66
2	Kinesiology	57
2	Schuessler salts	45
2	Hildegard medicine	41
2	Color therapy	38
2	Bach flowers	37
2	Reiki	30
2	Iris diagnostics	27

*Note.* Results from the first (n = 50) and second (n = 40) Delphi rounds. ^a^Seven-point Likert scale (7 = highly relevant, 1 = not at all relevant); ^b^Percentage of ratings on Likert scale 5–7 in Group 2, and Likert scale 4–7 in Group 3 and 4; ^c^Topics are assigned to Group 2 if at least 80% of the respondents gave a rating of 5–7 on the Likert scale; ^d^Topics are assigned to Group 3 if at least 80% of respondents gave a rating of 4–7 on the Likert scale; ^e^Topics are assigned to Group 4 if < 80% of respondents gave a rating of 4–7 on the Likert scale

### General remarks

**Fig. 2:**
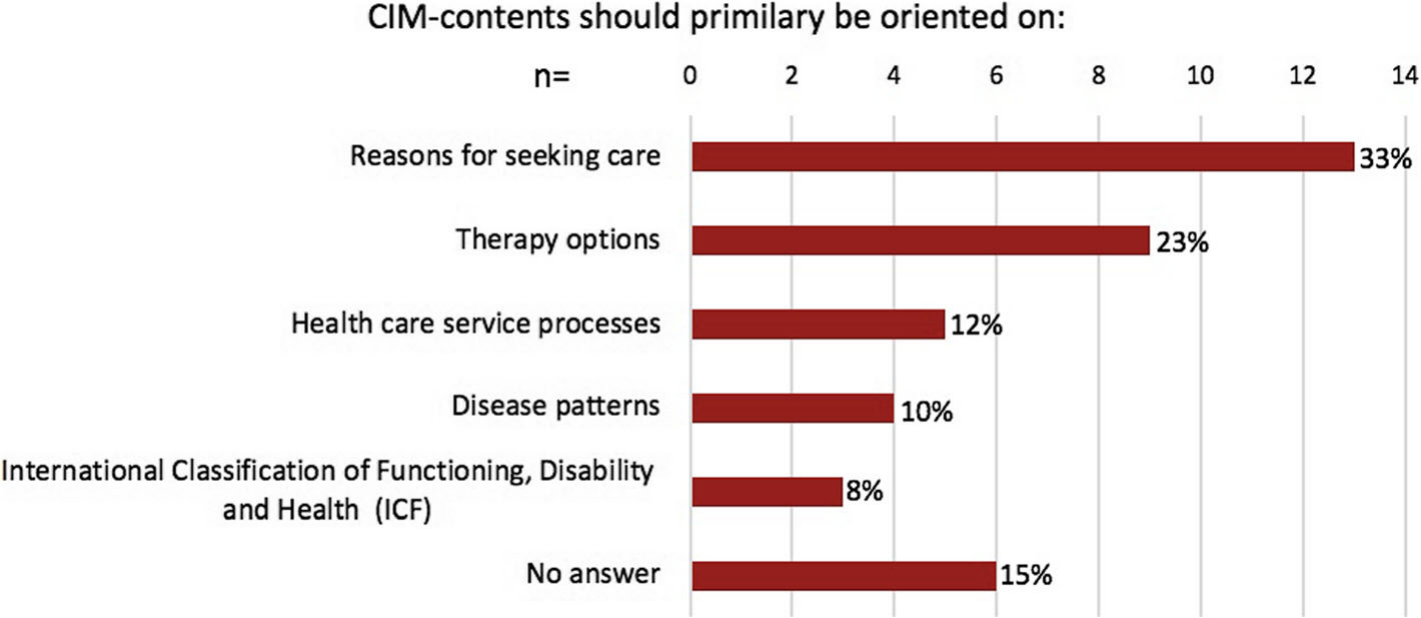
Orientation of CIM-contents in the curriculum. Question: How should the content of a CIM curriculum be structured? Multiple choice question; second Delphi round (n = 40)

In each Delphi round the free-text fields were used by the experts to make further suggestions or express criticism. In the first round, the criticism was expressed that the specific content should be aligned along therapeutic methods. In addition, some experts voiced that it would be important to address the combination of conventional procedures with non-conventional procedures to foster an integrative medical treatment approach in the teaching program. For this reason, in the second round a question was asked in response to which experts could indicate how the content of an interprofessional teaching program should be structured (Fig. 2).

In the second Delphi round, the experts were also asked to name treatment reasons which could be addressed in an interprofessional CIM seminar. The numerous suggestions were divided into 45 topics and arranged, according to the German National Catalogue of Competency-based Learning Objectives for Medicine (NKLM) [36] into *reasons for seeking care* (NKLM, chapter 20), *disease patterns* (NKLM, chapter 21) and *patient groups*. The wording in the NKLM was adopted for the terms by the main researcher. The 45 topics were submitted to the experts for evaluation in the third round. These assessments were also sorted into relevance groups (Table 4).

**Table 4:** Relevance groups for treatment reasons concerning CIM

Rating Question^a^ ***Please assess the topics in terms of their relevance for an interprofessional teaching program***	Consensus^b^
**Group 1: Highly relevant**^**c**^	
Reasons for seeking care	
Fatigue, exhaustion, general weakness	83
Generalized pain, pain at multiple localizations	81
Insomnia	81
Back pain	80
Disease patterns	
Irritable bowel syndrome	80
**Group 2: Relevant**^**d**^	
Reasons for seeking care	
Headache	90
Fear and anxiety	84
Pain in the extremities and joints	84
Complaints without identifiable physical cause	83
Disease patterns	
Chronic back pain	93
Burnout	90
Migraine	86
Depression	83
Menopause, climacteric (menopause)	83
Anxiety disorders	80
Patient groups	
Oncology, survivors: Support for cancer-related fatigue	93
Oncological patients: Support during radiation	81
Oncological patients: Support after surgery	80
Oncological patients: Support for complaints caused by chemotherapy	80
**Group 3: Partially relevant**^**e**^	
Reasons for seeking care	
Abnormal menstrual periods and cycle irregularities	87
Disease patterns	
Obesity	94
Metabolic syndrome	89
Fibromyalgia syndrome	87
Atopic eczema/neurodermatitis	84
Chronic and acute bronchitis	84
Arthrosis	83
Chronic inflammatory intestinal diseases	83
Essential hypertension	82
Bronchial asthma	81
Arthritis (inflammation of the joints)	81
Infections of the kidney and urinary tract (e.g. cystitis)	80
Diabetes mellitus type 2	80
Patient groups	
Healthy: Support for the development of self- and health competence	90
**Group 4: Not relevant**^**f**^	
Reasons for seeking care	
Cough	77
Fever	71
High-risk pregnancy and pregnancy problems like hyperemesis (vomiting)	70
Sore throat	69
Pregnancy (without known symptoms)	61
Disease patterns	
Allergic rhino conjunctivitis/allergic rhinosinusitis (hay fever)	72
Diabetes mellitus type 1	61
Patient groups	
Pediatrics: Pediatric diseases	76
Puerperium: Support during breastfeeding, milk congestion	76
Puerperium: Support for regression	75
Injuries and wounds: Support of the healing process	74
Healthy: Support for susceptibility to infection	74

*Note.* Results from the third Delphi round (*n* = 36). ^a^Seven-point Likert scale (7 = very relevant, 1 = not at all relevant); ^b^Percentage of ratings on Likert scale 6–7 in Group 1, 5–7 in Group 2, and Likert scale 4–7 in Group 3 and 4; ^c^Topics are assigned to Group 1 if at least  80% of the respondents gave a rating of 6–7 on the Likert scale; ^d^Topics are assigned to Group 2 if at least 80% of respondents gave a rating of 4–7 on the Likert scale; ^e^Topics are assigned to Group 3 if at least 80% of respondents gave a rating of 4–7 on the Likert scale; ^f^Topics are assigned to Group 4 if < 80% of respondents gave a rating of 4-7 on the Likert scale.

### Final workshop

The following points concerning topics for interprofessional education on CIM were highlighted in the final workshop:

- A focus could be placed on clinical guidelines because these highlight evidence for recommended CIM therapies.
- For the complementary methods taught in the seminar, it would be important to clarify the evidence base. Sackett’s approach would be helpful here, in which, in addition to the scientific evidence, the demands and preferences of the patients and the experience and skills of the experts are also important [37]. Which criteria are important in the evaluation of individual procedures for safe and professional care could be discussed with the students.
- In case-based teaching, it would be easier to start with a diagnosis than with general symptoms because a correct diagnosis/differential diagnosis would first have to be made in order to avoid premature treatment. It may be possible, however, to address “nursing diagnoses” or accompanying symptoms of an already diagnosed disease, such as pain or constipation.

## Discussion

The *Delphi method* proved suitable for achieving a consensus among the different experts on relevant content for an interprofessional CIM seminar with 15 teaching lessons, 90 min each. The experts showed a high level of active participation in all three rounds. The response rate was high, especially considering that the survey process lasted over a year and answering the various questions was time-consuming. In addition, the experts actively used the free-text fields and provided valuable suggestions for the subsequent rounds. The general topics and therapy methods already proposed in the first Delphi round did not prevent the experts from identifying further options or from questioning the strong weighting of therapy methods in the first round. The three rounds provided enough time to take up and consider completely new suggestions, such as structuring the content according to treatment reasons. Nevertheless, the Delphi method also showed limitations, especially because content and methods could not be discussed in relation to each other in the written survey process. In this respect, the Delphi workshop proved to be a constructive way to discuss the results and achieve a group consensus.

Regarding the *general topics and therapy methods*, none of these could be assigned to the highly relevant group. However, classification in the relevance group 2 means that at least 80% of the respondents voted positively on a seven-point Likert scale (rating of 5–7) so that a consensus on the relevance of these topics could be found here as well. The experts assumed that it would not only be important for students to gain an overview of many methods and concepts, but also to be able to assess effects and side-effects. Other surveys also emphasize the importance of teaching basics such as CIM terms, evidence and reasons for using CIM [38, 39]. For interprofessional communication, the use of the same terminology is essential since communication plays a major role in complementary medicine, both for communicating with patients and between the different professional groups [40, 41]. It is especially important that the professionals use the same terms in the case of chronically ill patients who are treated by different professionals over a longer period of time. This common understanding is a prerequisite to ensure patient safety. As described in other studies, the focus of an interprofessional CIM seminar should not primarily be to provide students with skills for the application of CIM therapies that they can incorporate directly into patient care, but rather to serve a more general learning purpose in providing broad knowledge about patient healthcare choices and to enable them to advise and guide patients accordingly [42, 43]. The PROFILES document, a contemporary revision of the objectives of medical studies in Switzerland, also describes that students in undergraduate medical training should be enabled to “adopt a shared decision-making approach in establishing the management plan […] take into account an indication or request for complementary medicine” ([44] p.23).

When evaluating individual *therapy concepts and methods*, a relatively large number of topics are assigned to relevance group 4. This group contains all topics on which no agreement could be found of at least 80% neutral or positive evaluations on the Likert scale (rating of 4–7). However, this does not preclude the possibility that individual experts have classified these topics as very relevant. In particular, topics such as homeopathy [45] which lack evidence for proof of biological efficacy are discussed very controversially. For other methods in this relevance group, it can be assumed that they are virtually unknown to the experts and are therefore not considered relevant. When considering the procedures classified as relevant, it can be seen that only for classical natural methods has a consensus been reached on their relevance. These methods are traditionally anchored in the European cultural area and go back in part to the concept of the Kneipp cure, which rests on five main tenets: hydrotherapy, exercise, nutrition, herbalism and the balance of mind and body [46]. It is possible that the weighting would be different in other cultural areas where other traditional methods are used. Despite the high level of awareness and the good evidence base, these topics have not yet been addressed consistently in medical training in Germany [47]. At the same time, these methods are well suited to actively involve the patient in the care process which will become increasingly important in view of the changing healthcare system [48].

With regard to the proposed *treatment reasons*, it is remarkable that a consensus could be reached on relevance group 1 for general pain, back pain, fatigue, insomnia and irritable bowel syndrome. These symptoms share a high level of complexity, a high psychological burden for the patient, limited conventional treatment options, and the possibility that many different professions can contribute to care. In addition, corresponding clinical guidelines exist that include complementary therapy methods and applications that are usually carried out by other healthcare professionals, for example, massage and lymph drainage for breast cancer patients [5] or music and movement therapy for paralysis [49], which are primarily performed by physiotherapists and occupational therapists.

The results of the written Delphi survey were largely confirmed in the *final workshop*. Here the topic of clinical guidelines was taken up again, as the evidence for treatment recommendations has already been reviewed by expert committees and supplementary procedures are also considered. It was acknowledged that it is important to address the extensive concept of evidence, which considers not only the scientific evidence, but also the preferences of the patient and the experience of the practitioner [37]. Aveni et al. describe that different professionals use different strategies to forge opinions regarding CIM: physicians relied more on scientific evidence, while nurses and midwives were more influenced by personal experience [50]. The WHO supports the integration of traditional medicine into national health systems, whether or not these methods are based on theories, beliefs or experience and whether or not they can be explained ([1], p.17). A corresponding interprofessional seminar should therefore provide ample opportunity for discussions to assess the evidence base and for shared decision-making. By doing this, profession-specific cultures can be overcome. The difficulty faced by a single practitioner when monitoring all treatment options and the increasing importance of involving other professions in treatment decisions were also discussed [51]. Furthermore, it became clear in the workshop that the initial diagnosis is not suitable for the interprofessional teaching environment. The patient cases presented in the seminar should be preceded by a differential diagnostic clarification. Especially in the case of chronic and complex clinical pictures, the interprofessional consideration of the symptoms, the need for care and the supportive therapy options of an already existing and diagnosed disease seem to be valuable for a holistic view of the patient. So-called nursing diagnoses, for example, can raise greater awareness of the accompanying symptoms and also of the self-care capabilities of the patient [52].

In our opinion, this study is the first to systematically identify possible contents for an interprofessional CIM seminar. The results determined in this survey can be easily reconciled with the following Swiss PROFILES objectives for undergraduate medical education: informing students about patients’ use of CIM treatments and to enable them to discuss these options with patients, taking into account benefits and risks, as well as interactions [34, 44]. The results can also linked with the competence descriptions of the Academic Collaborative for Integrative Health [53] in which the following key competencies are described: interprofessional communication, teamwork, roles and responsibilities, values and ethics, as well as evidence-based work and the integration of all practitioners into the healthcare system. There are also general recommendations concerning interprofessional teaching methods which can also be linked to the results of this study [54, 55]. Early interprofessional learning based on concrete patient case studies is considered necessary for later team-based and patient-centered collaboration [19, 56]. Other current studies on interprofessional teaching on CIM also focus on interactive teaching methods, for example, simulation-based learning [57] or online courses for chronic disease management [58]. In the future, more research is needed on CIM education that focuses both on teaching methods and on comparing information and academic perspectives within and between CIM institutions and medical faculties [59].

### Limitation

This is a non-representative survey among experts with different professional backgrounds and experiences. Perhaps a different composition, for instance, with the inclusion of other health professions would have produced different results. Nevertheless, due to the open questions and active participation, it was possible to bring about a process-related development through the three Delphi rounds and to stimulate fruitful discussion. Consensus could be reached on much of the content. It might also have been interesting to discuss even more deeply the contents on which no consensus was reached. Concrete implementation of a curriculum depends on general conditions, such as the participants, temporal circumstances, and its embedding in the overall curriculum. Therefore, these results can only be viewed as suggestions for development and refinement of interprofessional CIM seminars and would need to be adapted accordingly for implementation.

## Conclusions

Overall, the results indicate that the focus of an interprofessional CIM seminar should initially be on imparting broad basic knowledge of CIM and familiarizing students with various treatment options. Conveying the content in a way that is patient-oriented is a promising strategy to promote communication and make the seminar application-oriented and practical. The concrete suggestions identified by the survey can be used as a good starting point to address student interests and find a high level of acceptance among lecturers and medical faculties.

## Supplementary Information

**Additional file 1:.** Supplementary file: Translated questionnaires. 1st – 3rd Delphi rounds.

## Data Availability

The datasets used and/or analyzed during the current study are available from the corresponding author on reasonable request.

## Abbreviations

CIM: Complementary and Integrative Medicine
NKLM: National Competency-Based Learning Objectives Catalogue for Medicine

## References

[1] World Health Organization (2013). WHO traditional medicine strategy: 2014–2023.

[2] Kemppainen LM, Kemppainen TT, Reippainen JA, Salmenniemi ST, Vuolanto PH (2018). Use of complementary and alternative medicine in Europe: health-related and sociodemographic determinants. Scand J Public Health.

[3] Deng GE, Frenkel M, Cohen L, Cassileth BR, Abrams DI, Capodice JL (2009). Evidence-based clinical practice guidelines for integrative oncology: complementary therapies and botanicals. J Soc Integr Oncol.

[4] Klose P, Kraft K, Cramer H, Lauche R, Dobos G, Langhorst J (2014). Phytotherapy in the German medical AWMF S3 guidelines - a systematic overview. Forsch Komplementmed.

[5] Greenlee H, DuPont-Reyes MJ, Balneaves LG, Carlson LE, Cohen MR, Deng G (2017). Clinical practice guidelines on the evidence-based use of integrative therapies during and after breast cancer treatment. CA Cancer J Clin.

[6] Hunter J, Leach M, Braun L, Bensoussan A (2017). An interpretive review of consensus statements on clinical guideline development and their application in the field of traditional and complementary medicine. BMC Complement Altern Med.

[7] Ni X, Lin H, Li H, Liao W, Luo X, Wu D (2020). Evidence-based practice guideline on integrative medicine for stroke 2019. J Evid Based Med.

[8] Deng GE, Rausch SM, Jones LW, Gulati A, Kumar NB, Greenlee H (2013). Complementary therapies and integrative medicine in lung cancer: diagnosis and management of lung cancer, 3rd ed: American College of Chest Physicians evidence-based clinical practice guidelines. Chest..

[9] Sanft T, Denlinger CS, Armenian S, Baker KS, Broderick G, Demark-Wahnefried W (2019). NCCN guidelines insights: survivorship, version 2.2019. J Natl Compr Cancer Netw.

[10] Foley H, Steel A, Cramer H, Wardle J, Adams J (2019). Disclosure of complementary medicine use to medical providers: A systematic review and meta-analysis. Sci Rep.

[11] White A, Boon H, Alraek T, Lewith G, Liu J-P, Norheim A-J (2014). Reducing the risk of complementary and alternative medicine (CAM): challenges and priorities. EuJIM..

[12] Hall H, Brosnan C, Frawley J, Wardle J, Collins M, Leach M (2018). Nurses’ communication regarding patients’ use of complementary and alternative medicine. Collegian..

[13] Mwaka AD, Tusabe G, Orach Garimoi C, Vohra S (2018). Turning a blind eye and a deaf ear to traditional and complementary medicine practice does not make it go away: a qualitative study exploring perceptions and attitudes of stakeholders towards the integration of traditional and complementary medicine into medical school curriculum in Uganda. BMC Med Educ.

[14] Wong LY, Toh MP, Kong KH (2010). Barriers to patient referral for complementary and alternative medicines and its implications on interventions. Complement Ther Med.

[15] Onal O, Sahin DS, Inanc BB (2016). Should CAM and CAM training programs be included in the curriculum of schools that provide health education?. Aust J Pharm.

[16] Loh KP, Ghorab H, Clarke E, Conroy R, Barlow J (2013). Medical students' knowledge, perceptions, and interest in complementary and alternative medicine. J Altern Complement Med.

[17] Teixeira Medeiros N, Fontenelle Catrib AM, Anchieta Mendes Melo N, Pessoa Marinho Holanda G, de Mesquita Martins LV, Pereira da Silva Godinho CC (2019). Academic education in health profession programs, knowledge and use of Complementary and Alternative Medicine (CAM) by university students. Complement Ther Med.

[18] Templeman K, Robinson A, McKenna L (2016). Advancing medical education: connecting interprofessional collaboration and education opportunities with integrative medicine initiatives to build shared learning. J Complement Integr Med.

[19] Rivera J, de Lisser R, Dhruva A, Fitzsimmons A, Hyde S, Reddy S (2018). Integrative health: an interprofessional standardized patient case for prelicensure learners. MedEdPORTAL..

[20] Academic Consortium for Integrative Medicine & Health (2018). Definition of integrative medicine and health.

[21] Interprofessional Education Collaborative Expert Panel (2016). Core competencies for interprofessional collaborative practice: 2016 update.

[22] CAIPE (2002). Centre For The Advancement Of Interprofessional Education.

[23] Kutt A, Mayan M, Bienko I, Davies J, Bhatt H, Vohra S (2019). An undergraduate course combining interprofessional education and complementary health approaches learning objectives: Successful integrative learning that improves interest and reduces redundancy. explore (NY).

[24] Haramati A, Adler SR, Wiles M, Sierpina VS, Kreitzer MJ (2013). Innovation and collaboration: The first international congress for educators in complementary and integrative medicine. Explore (New York, NY).

[25] Kutt A, Mayan M, Bienko I, Davies J, Bhatt H, Vohra S (2019). An undergraduate course combining interprofessional education and complementary health approaches learning objectives: Successful integrative learning that improves interest and reduces redundancy. Explore (NY).

[26] Willison KD (2008). Advancing integrative medicine through interprofessional education. Health Sociol Rev.

[27] Geigle PR, Galantino ML (2009). Complementary and alternative medicine inclusion in physical therapist education in the United States. Physiother Res Int.

[28] Gold J, Anastasi J (1995). Education opportunities in alternative/complementary medicine for nurses. J Altern Complement Med.

[29] Linstone HA, Turoff M (2002). The Delphi method: techniques and applications: Addison-Wesley publishing company.

[30] Diamond IR, Grant RC, Feldman BM, Pencharz PB, Ling SC, Moore AM (2014). Defining consensus: a systematic review recommends methodologic criteria for reporting of Delphi studies. J Clin Epidemiol.

[31] Nicolao M, Tauber MG, Heusser P (2010). How should complementary and alternative medicine be taught to medical students in Switzerland? A survey of medical experts and students. Med Teach.

[32] Joos S, Eicher C, Musselmann B, Kadmon M (2008). Development and implementation of a ‘curriculum complementary and alternative medicine’ at the Heidelberg medical school. Forsch Komplementmed..

[33] Owen D, Lewith GT (2001). Complementary and alternative medicine (CAM) in the undergraduate medical curriculum: the Southampton experience. Med Educ.

[34] Stuttard P, Walker E (2000). Integrating complementary medicine into the nursing curriculum. Complement Ther Nurs Midwifery.

[35] Turtuff M, Linstone HA, Turoff M (2002). The Policy Delphi. The Delphi method: Techniques and applications: New Jersey Institute of Technology.

[36] NKLM (2015). Nationaler Kompetenzbasierter Lernzielkatalog Medizin [National Competency-Based Learning Objective Catalogue].

[37] Sackett DL, Rosenberg WMC, Gray JAM, Haynes RB, Richardson WS (1996). Evidence based medicine: what it is and what it isn’t. BMJ..

[38] Maharaj SR (2010). Preparing medical graduates to practise in a changing environment: complementary/alternative medicine in the medical undergraduate curriculum of the University of the West Indies. West Indian Med J.

[39] Klafke N, Homberg A, Glassen K, Mahler C (2016). Addressing holistic healthcare needs of oncology patients: implementation and evaluation of a complementary and alternative medicine (CAM) course within an elective module designed for healthcare professionals. Complement Ther Med..

[40] Nguyen J, Smith L, Hunter J, Harnett JE (2019). Conventional and complementary medicine health care practitioners’ perspectives on interprofessional communication: A qualitative rapid review. Medicina (Kaunas).

[41] Frenkel M, Cohen L (2014). Effective communication about the use of complementary and integrative medicine in cancer care. J Altern Complement Med.

[42] Cowen VS, Cyr V (2015). Complementary and alternative medicine in US medical schools. Adv Med Educ Pract.

[43] Kreitzer MJ, Mann D, Lumpkin M (2008). CAM competencies for the health professionals. Complement Health Pract Rev.

[44] Michaud PA, Jucker-Kupper P, members of the Profiles working group (2017). PROFILES; Principal Objectives and Framework for Integrated Learning and Education in Switzerland.

[45] Rutten L, Mathie RT, Fisher P, Goossens M, van Wassenhoven M (2013). Plausibility and evidence: the case of homeopathy. Med Health Care Philos.

[46] Ko Y (2016). Sebastian Kneipp and the natural cure movement of Germany: between naturalism and modern medicine. Uisahak..

[47] Stock-Schroeer B, Huber R, Joos S, Klose P (2017). Evaluation of the current status of rehabilitation, physical medicine and naturopathy education 10 years after the reform of the Medical Licensure Act - a nationwide survey of German Medical Universities. GMS J Med Educ.

[48] Barnett H (2017). Complementary and alternative medicine and patient choice in primary care. Qual Prim Care.

[49] DGNR-LL (2020). AWMF-Register No. 080/003 [S3 Guideline “Rehabilitative therapy for arm paresis after stroke”]. AWMF.

[50] Aveni E, Bauer B, Ramelet AS, Decosterd I, Ballabeni P, Bonvin E (2017). Healthcare professionals' sources of knowledge of complementary medicine in an academic center. PLoS One.

[51] Green BN, Johnson CD (2015). Interprofessional collaboration in research, education, and clinical practice: working together for a better future. J Chiropr Educ.

[52] Brokel JM (2016). Nurses and nurse practitioners diagnose to plan evidence-based care and treatments. Int J Nurs Knowl.

[53] Academic Collaborative for Integrative Health. Competencies for optimal practice in integrated environments. In: ACIH Board of Directors, editor. Los Angeles; 2018.

[54] Bridges DR, Davidson RA, Odegard PS, Maki IV, Tomkowiak J. Interprofessional collaboration: three best practice models of interprofessional education. Med Educ Online [Internet]. 2011:16. Available from: https://www.ncbi.nlm.nih.gov/pubmed/21519399.A. 10.3402/meo.v16i0.6035 PMC3081249 21519399

[55] Berger S, Goetz K, Leowardi-Bauer C, Schultz JH, Szecsenyi J, Mahler C. Anchoring interprofessional education in undergraduate curricula: the Heidelberg story. J Interprof Care. 2017;31(2):175–9. 10.1080/13561820.2016.1240156 27880080

[56] Barr H, Ford J, Gray R, Helme M, Hutchings M, Low H, et al. Interprofessional education guidelines: CAIPE: Centre for the Advancement of Interprofessional Education; 2017. Available from: https://www.caipe.org/news/guidance-on-global-interprofessional-education-and-collaborative-practice-research.

[57] Cunningham S, Foote L, Sowder M, Cunningham C (2018). Interprofessional education and collaboration: a simulation-based learning experience focused on common and complementary skills in an acute care environment. J Interprof Care..

[58] McCabe C, Patel KD, Fletcher S, Winters N, Sheaf G, Varley J, et al. Online interprofessional education related to chronic illness for health professionals: a scoping review. J Interprof Care [Internet]. 2020:1-10. Available from: https://www.ncbi.nlm.nih.gov/pubmed/32323605. 10.1080/13561820.2020.1749575 32323605

[59] Gray AC, Steel A, Adams J. Attitudes to and uptake of learning technologies in complementary medicine education: results of an international faculty survey. J Altern Complement Med [Internet]. 2020. Available from: https://www.ncbi.nlm.nih.gov/pubmed/32013531. 10.1089/acm.2019.0319 32013531

