# Supplementary material for: Findings from a three-round Delphi study: essential topics for interprofessional training on complementary and integrative medicine
Source: BMC Complement Med Ther. 2020 Nov 17;20:348. doi: 10.1186/s12906-020-03140-x (PMC7670669; doi:10.1186/s12906-020-03140-x)
Supplement: Supplementary file 1 — Additional file 1:. Supplementary file: Translated questionnaires. 1st – 3rd Delphi rounds. [file 12906_2020_3140_MOESM1_ESM.pdf]

Findings from a Three-Round Delphi Study: Essential Topics for Interprofessional Training on Complementary and Integrative Medicine

Angelika Homberg, Nadja Klafke, Svetla Loukanova, Katharina Glassen

Supplementary file, Translated questionnaires

1st Delphi Round: Introduction

Delphi Study: Curriculum Development for Interprofessional Education on Complementary and Integrative Medicine

First Round

We welcome you to the first round of the Delphi survey "Development of an interprofessional curriculum on complementary and integrative medicine"

Thank you very much for participating in this survey. When answering the questions, please refer to a potential seminar for **C**omplementary and **I**ntegrative **M**edicine (CIM) with 30-60 teaching units of 45 min each, which is offered at a medical school for students of medicine and other healthcare professions (from the fields of nursing, therapy and diagnostics) in undergraduate education. This survey is meant to record your personal opinion on and assessment of contents and objectives for such a curriculum. Please fill in the questionnaire as completely as possible and use the free text to make comments and further additions. The survey will be conducted pseudonymously. Your evaluations and verbatim core statements from the free text answers will be included in the next survey round and in the result report, but it will not be possible to draw conclusions about your identity.

In addition to the socio-demographic surveys, the survey includes one section on the addressed goals and competencies and one section on the possible curricular content.

It should take about 20 to 30 minutes to complete the questionnaire.

You can stop and come back to continue the questionnaire at a later time. Your results will be stored temporarily.

If you have any questions, please do not hesitate to contact me.

Angelika Homberg
Phone +49 (0) 6221 567169
angelika.homberg@med.uni-heidelberg.de

NEXT

1st Delphi Round: Socio-demographic Data (Part A)

How old are you? ☐

Gender
- female ☐
- male ☐

To which profession group do you belong? (Multiple answers possible)
- Nursing ☐
- Therapy ☐
- Medical technology/diagnostics ☐
- Medicine ☐
- Pharmacy ☐
- Social Work/Social Pedagogics ☐
- Sports Science ☐
- Educational Science ☐
- Midwifery ☐
- Psychology ☐

Others ____________________

Which professional and/or academic degree do you have? ____________________

What additional qualifications do you have? ____________________

Current fields of activity (multiple answers possible)
- Student ☐
- Inpatient care ☐
- Outpatient care ☐
- Rehabilitation ☐
- Prevention ☐
- Patient counseling ☐
- Oncology ☐
- Pediatrics/child care ☐
- Geriatrics/geriatric care ☐
- Palliative care ☐
- Teaching at the college, university ☐
- Apprenticeship at the university of applied sciences, vocational school ☐
- Research ☐

Others ____________________

What is your current focus of activity? ____________________

In which country/state are you mainly working?
- Baden-Württemberg ☐
- Bavaria ☐
- Berlin ☐
- Brandenburg ☐
- Bremen ☐
- Hamburg ☐
- Hesse ☐
- Mecklenburg-Western Pomerania ☐
- Lower Saxony ☐
- North Rhine-Westphalia ☐
- Rhineland-Palatinate ☐
- Saarland ☐
- Saxony ☐
- Saxony-Anhalt ☐
- Schleswig-Holstein ☐
- Thuringia ☐
- Austria ☐
- Switzerland ☐

Other ____________________

Do you have a pedagogical qualification?
- No ☐
- Medical-didactic training, pedagogical seminar < 10 teaching hours ☐
- Medical-didactic training, pedagogical seminar 10-99 teaching hours (e.g. lecturer training) ☐
- Medical-didactic training, pedagogical seminar > 100 teaching hours ☐
- Specific training / study (e.g. Master of Medical Education) ☐

Others ____________________

Do you have experience in academic teaching? (multiple answers possible)

No, no teaching experience at all	
No, teaching experience only in non-academic education	
Less than 15 lessons (á 45 min) per semester during the last year	
Performing 15 or more lessons (á 45 min) per semester in the last year	
Teaching experience for less than five years	
Teaching experience for five or more years	
Experience in interprofessional teaching	
Experience in teaching coordination	
Experience in curriculum development	

What is your practical professional experience in the following areas of patient care? (including internships and periods of further training, multiple answers possible)

	no experiene	< 3 years	3-8 years	> 8 years	no answer
Inpatient sector					
Outpatient sector					
Prevention					
Rehabilitation					
Palliative care					
Patient consultation					

What is your professional experience in the following research areas?

	no experiene	< 3 years	3-8 years	> 8 years	no answer
Basic Research					
Clinical research					
Health Services Research					
Training research					
Further research areas					

How many years of professional experience do you have in total?

What is your basic attitude towards complementary medicine? Please rank yourself on the scale of 1-10 listed here.

very negativ									very positiv

If you use complementary medicine methods in a professional context, please indicate here the generic terms or the classification of the respective methods (e.g. phytotherapy, TCM, Ayurveda ...).

NEXT

1st Delphi Round: Competencies (Part B)

When answering the questions, please refer to a potential seminar for Complementary and Integrative Medicine (CIM) with 30-60 teaching units of 45 min each, which is offered at a medical school for students of medicine and other healthcare professions (from the fields of nursing, therapy and diagnostics) in undergraduate education.

Below you will find a list of teaching-learning objectives and competencies based on CIM curricula already described in the literature. Please rate them with regard to their relevance on a seven-point Likert scale. Please also indicate whether these competencies should be addressed explicitly or implicitly ("between the lines").

Use the free text to justify and supplement your assessment and to list further competencies.

Your comments will be will be listed anonymously in the next survey round. The assessed competencies will be assigned to four relevance groups based on the assessments.

How should the competencies be addressed?

Teaching objectives and competencies (CIM = complementary and integrative medicine)	1=not at all relevant	2	3	4	5	6	7=very relevant	rather explicitly	rather implicitly
B1.1 Students are able to classify CIM terms and methods correctly.									
B1.2 Students are able to form their own opinion about the respective procedures.									
B1.3 Students are able to reflect and explain their beliefs and attitudes towards CIM.									
B1.4 Students are able to expand their knowledge of CIM procedures.									
B1.5 Students are able to assess CIM methods with regard to their evidence on the basis of corresponding literature research.									
B1.6 Students are able to assess CIM methods with regard to their evidence on the basis of corresponding literature research.									
B1.7 Students are able to critically evaluate publications on CIM.									
B1.8 Students are able to independently apply selected methods to patient care.									
B1.9 Students are able to recognize their own possibilities and limitations in the interprofessional healthcare team.									

	1=not at all relevant	2	3	4	5	6	7=very relevant	rather explicit	rather implicit
B1.10 Students are able to be familiar with the skills of all professions involved in CIM care.									
B1.11 Students are able to discuss CIM in the interprofessional healthcare team.									
B1.12 Students are able to recognize how their specific occupational roles contribute to provide the necessary patient care when working in interprofessional teams.									
B1.13 Students are able to respect different therapeutic perspectives and practices									
B1.14 Students are able to inquire and recognize the physical, psychological and social care needs of a patient.									
B1.15 Students are able to respect and consider patient perspectives and subjective experiences of illness.									
B1.16 Students are able to show openness to the wishes and attitudes of the patient and treat them respectfully.									
B1.17 Students are able to advise patients and relatives on CIM in an interprofessional team.									
B1.18 Students are able to handle their own health carefully with awareness.									

Select up to three competencies from the following lists that seem most important to you	Drop-down-Menu: B1.1-B1.18
Here you can justify the assessments you have made and/or list which aspects of the listed content you consider particularly important.Please indicate the corresponding number (B1.1-B1.18).	
Which other competencies do you consider relevant?<br>The newly mentioned competencies will be submitted for evaluation inte next survey round on a seven point likert scale.	

NEXT

1st Delphi Round: CanMEDS Role Cometencies (Part C)

Competencies in medical education are nationally and internationally oriented to the CanMEDS. This competency model was developed for physicians by the Royal College of Physicians and Surgeons of Canada in 2005 and is based on different roles that can be assumed by the respective professions in healthcare. Many curricula, including the National Competency-based Learning Objectives Catalogue of Medicine (NKLM), are based on this competence framework.

First assess which of the listed sub-competencies of the role of the collaborator are relevant in interprofessional CIM care.

	1=not at all relevant	2	3	4	5	6	7=very relevant
C1.1 Graduates participate actively and constructively in the teamwork for the joint accomplishment of tasks.							
C1.2 Graduates develop concepts, guidelines, treatment pathways, etc. in cooperation with other healthcare professions which can support and further the quality of care.							
C1.3 Graduates behave in an appreciative manner in interprofessional cooperation and thus contribute to good patient care.							
C1.4 Graduates have a professional role identity and know the roles, competencies and responsibilities of the other professional groups involved.							
C1.5 Graduates recognise interprofessional conflicts and actively contribute to productive and appropriate solutions.							
C1.6 Graduates explain their role identity (tasks and duties) to others.							
C1.7 Graduates reflect their tasks with regard to continuous patient care.							
C1.8 Graduates work together with various health professions and disciplines in an appropriate, appreciative and efficient manner.							
C1.9 Graduates analyse existing healthcare structures when interacting with other healthcare professions and evaluate them in terms of their effectiveness and efficiency.							
C1.10 Together with the other team members, graduates evaluate the quality of the joint work and, if necessary, agree on measures for improvement.							

Which of the other listed CanMEDS roles do you consider relevant for all academic health care professions in the context of interprofessional CIM care? The CanMEDS roles classified as relevant will be broken down and submitted again in the next survey round for evaluation (80% > scale level 5).

	1=not at all relevant	2	3	4	5	6	7=very relevant
C2.1 Scholar: Readiness for lifelong learning, application and dissemination of medical knowledge							
C2.2 Communicator: Establishing a positive patient relationship, if necessary with the involvement of family members and professions involved.							
C2.3 Health Advocate: Advocating for the needs and satisfaction of patients, communities and population groups							
C2.4 Leader: Careful use of resources, contribution to increasing effectiveness in the healthcare system							
C2.5 Professional: Selfless attitude, ethical decision-making, assumption of responsibility							
C2.6 Medical Expert: Integration of all CanMEDS roles, knowledge, skills and attitudes support patient-centred care							

Here you can justify the assessments made on the CanMEDS roles and/or list which aspects you consider particularly important. Please indicate the corresponding competency number.

NEXT

1st Delphi Round: Curricular Content (Part D)

In the following, some teaching content are listed. Please assess their relevance for an interprofessional CIM curriculum on a seven-point Likert scale. In addition, you have the possibility to indicate if you cannot or do not want to assess the content due to lack of knowledge and/or for other reasons.

Please use the free-text fields to justify or supplement your assessments and to list further possible teaching content.

Your comments will be listed in the next survey round. The newly listed content will be submitted for evaluation in the second round of questioning. The assessed content will be assigned to four relevance groups (very relevant - not relevant). You will then have the opportunity to agree or disagree with the assignment and to add further comments.

	1=not at all relevant	2	3	4	5	6	7=very relevant	I feel not competent to assess
D1.1 Historical and philosophical backgrounds of CIM therapy								
D1.2 Clarification and explanation of CIM terms (natural medicine, complementary and alternative medicine, ...)								
D1.3 Legal issues relating to delegation, substitution and reserved activities for certain professions within CIM patient care								
D1.4 Use of CIM therapies								
D1.5 Supply structures and service providers (alternative practitioners, doctors with additional qualifications, health professions, CIM facilities)								
D1.6 Overview of CIM databases and literature search via the Internet								
D1.7 Deepening the assessment of CIM evidence on the basis of selected studies								
D1.8 Overview of effects and interactions of selected CIM therapies								
D1.9 Overview of methods in classical natural medicine (exercise, nutrition, hydrotherapy, herbalism and the balance of mind and body)								
D1.10 Overview of methods in non-classical natural medicine (e.g. Acupuncture, Homeopathy, Anthroposophical medicine)								

Here you can justify the assessments you have made and/or list which aspects of the listed content you consider particularly important. Please indicate the corresponding number (D.1.1-D.1.10).

Here you can list further general content for an interprofessional CIM

Classical natural medicine

The classical natural medicine methods classified as relevant will be broken down in detail in the next survey round and submitted again for evaluation (80% > scale level 5).

	1=not at all relevant	2	3	4	5	6	7=very relevant	I feel not competent to assess
D2.1 Overview: Hydrotherapy (Bath, wraps, pourings)								
D2.2 Overview: Regulative therapy / Mind-Body medicine (Yoga, Tai chi, Qui gong, progressive muscle relaxation)								
D2.3 Overview: Movement therapy (Exercise training, walking)								
D2.4 Overview: Dietetics (nutritional recommendations, high-dose vitamins, trace elements)								
D2.5 Overview: Phytotherapy (medicinal plants, teas, aromatherapy, herbal medicines)								

Here you can justify the assessments you have made and/or list which aspects of the listed content you consider particularly important. Please indicate the corresponding number (D.2.1-D.2.5).

Non-classical natural medicine

	1=not at all relevant	2	3	4	5	6	7=very relevant	I feel not competent to assess
D3.1 Neural therapy								
D3.2 Chiropractic								
D3.3 Cranio-sacral therapy								
D3.4 Homeopathy								
D3.5 Acupuncture								
D3.6 Traditional Chinese Medicine (TCM)								
D3.7 Anthroposophical medicine								
D3.8 Ayurveda								
D3.9 Tibetan medicine								
D3.10 Diverting procedures (leeches, cupping)								

Here you can justify the assessments you have made and/or list which aspects of the listed content you consider particularly important. Please indicate the

Which other non-classical medicine methods do you consider relevant?

You have reached the end of the interview. Is there anything else you'd like to

SUBMIT

Delphi Study: Curriculum Development for Interprofessional Education on Complementary and Integrative Medicine

Second Round

We welcome you to the second round of the Delphi survey "Development of an interprofessional curriculum on complementary and integrative medicine"

Thank you very much for participating in the first survey round. The present questionnaire was designed on the basis of your weightings, suggestions and constructive criticism. All comments were fully incorporated into the second survey wave. A compilation of all quotations can be found in the attached result report.
Part A of this second survey contains questions on teaching methods, Part B: questions on competencies, Part C: questions on role competencies and Part D: questions on curricular content.

When answering the questions, please refer to a potential seminar for Complementary and Integrative Medicine (CIM) with 30-60 teaching units of 45 min each, which is offered at a medical school for students of medicine and other healthcare professions (from the fields of nursing, therapy and diagnostics) in undergraduate education.
This survey is meant to record your personal opinion on and assessment of contents and objectives for such a curriculum. Please fill in the questionnaire as completely as possible and use the free text to make comments and further additions. The survey will be conducted pseudonymously. Your evaluations and verbatim core statements from the free text answers will be included in the next survey round and in the result report, but it will not be possible to draw conclusions about your identity.

It should take about 30 minutes to complete the questionnaire.

You can stop and come back to continue the questionnaire at a later time. Your results will be stored temporarily.

If you have any questions, please do not hesitate to contact me.

Angelika Homberg
Phone +49 (0) 6221 567169
angelika.homberg@med.uni-heidelberg.de

NEXT

2nd Delphi Round: Teaching Methods (Part A)

In the first survey round, some methodological-didactic aspects for an interprofessional complementary and integrative medical curriculum were taken up. Since the development of a teaching and learning program cannot be carried out without without taking teaching methods into consideration (assessment drives learning), your comments and suggestions from the first survey wave will be taken up and discussed at the beginning of this survey wave.

When answering the questions, please refer to a potential seminar for Complementary and Integrative Medicine (CIM) with 30-60 teaching units of 45 min each, which is offered at a medical school for students of medicine and other healthcare professions (from the fields of nursing, therapy and diagnostics) in undergraduate education.

From the following list, select the learning setting that you think is best suited for an interprofessional CIM seminar!
(CIM=Complementary and integrative medicine)
Please choose one of the following answers!

- [ ] CIM should be offered as a curricularly anchored elective module for all students in the medical and health professions.
- [ ] CIM should be offered as a compulsory basic module for all students in the medical and health professions in the curriculum of the main study programme.
- [ ] CIM basic knowledge should be offered as a compulsory module for all students in the medical and health professions. In addition, there should be the opportunity to deepen individual areas in elective modules.
- [ ] CIM should be offered voluntarily, extracurricular modules with certificate degree.
- [ ] CIM should not be offered as an independent interprofessional module but integrated more strongly into existing modules.
- [ ] CIM should not be taught at universities and colleges.

Others ______
- [ ] No answer

Select from the following list the structural orientation of the contents of an interprofessional CIM curriculum that best suits your needs.
Please choose one of the following answers!

- [ ] CIM-contents should primilary be oriented on therapy options.
- [ ] CIM-contents should primilary be oriented on disease patterns.
- [ ] CIM-contents should primilary be oriented on reasons for seeking care.
- [ ] CIM-contents should primilary be oriented on health care service processes.
- [ ] CIM-contents should primilary be oriented on International Clssification of Functioning, Disability and Health (ICF).

Others ______
- [ ] No answer

Please select from the following list for which fields of activity an interprofessional CIM seminar should primarily qualify.
If you think the statement is correct, please tick the box on the left.
If the statement is only partially true or under certain conditions, you can write a comment on the right.

	I agree	Free-text field
Interprofessional CIM care is relevant for the acute inpatient sector.		
Interprofessional CIM care is relevant for the chronic-rehabilitative area.		
Interprofessional CIM care is relevant for the outpatient sector.		
Interprofessional CIM care is relevant to the field of prevention and health promotion.		
Interprofessional CIM care is relevant for the support of self- and lay therapy and care.		
Interprofessional CIM care cannot be implemented in the (present and future) care reality and is therefore not relevant.		

Others ______

Please select from the following list which teaching methods should be primarily used in a CIM curriculum.
If you think the statement is correct, please tick the box on the left.
If the statement is only partially true or under certain conditions, you can write a comment on the right.

	I agree	Free-text field
CIM content/knowledge should be presented in a structured way in the classical lecture format.		
CIM content should be presented as complex case studies (case-oriented learning).		
CIM knowledge should be acquired by self-directed learninig (problem-oriented learning).		
Students shoud discuss ethical questions and contradictory statements regarding CIM.		
Students should share their own experiences, opinions and attitudes with regard to CIM.		
Students shoud try out simple procedures and CIM methods for themselves (experience-oriented learning).		
Real patients and/or acting patients should be involved in the seminars as often as possible.		

Others ______

NEXT

2nd Delphi Round: Competencies (Part B)

In the first wave of the survey, competencies were assessed on a seven-point Likert scale (1=not at all relevant to 7=very relevant). In the evaluation these were classified into the following four categories:

highly relevant (at least 80% of the respondents rated it with a 6 or 7 on the Likert scale)
relevant (at least 80% of the respondents rated it with a 5-7 on the Likert scale)
partly relevant (at least 80% of the respondents rated it with a 4-7 on the Likert scale)
not relevant (remaining topics)
Furthermore, the free-text fields were evaluated and hypotheses were derived. The hypotheses and the newly listed competencies are now presented to you for evaluation.

In the following, competencies are arranged in descending hierarchical order according to the relevance assessment already carried out, but retains its original numbering from the first survey wave. Please indicate whether you agree with the groupings and hypotheses.
If you fully agree with the statement, please tick the respective box on the left side of the page.
If you do not agree or only partially agree with the statement or would like to add something, please use the free text field behind the statement.

	I agree	Free-text field
B1.16 The competency "**Students are able to show openness to the wishes and attitudes of the patient and treat them respectfully**" is very relevant as a cornerstone of evidence-based medicine even independently of CIM and forms the basis of all therapeutic action.		
B1.3 The competency "**Students are able to reflect and explain their beliefs and attitudes towards CIM**" is very relevant and should be explicitly taught in a CIM curriculum.		
B1.15 The competency "**Students are able to respect and consider patient perspectives and subjective experiences of illnes**" is very relevant independently of CIM. However, students should already have knowledge of treatment methods and therapy options.		
B1.1 The competency **"Students are able to classify CIM terms and methods correctly"** is relevant and, as a solid knowledge base, is a prerequisite for the other contents of the CIM curriculum. This competence should therefore be taught explicitly.		
B1.2 The competency "**Students are able to form their own opinion about the respective procedures**" is relevant, but the formation of opinion should be based on scientific principles.		
B1.11 The competency "**Students are able to discuss CIM in the interprofessional healthcare team**" is relevant, but should be taught rather implicitly.		
B1.9 The competency "**Students are able to recognize their own possibilities and limitations in the interprofessional healthcare team**" is relevant and serves to avoid exceeding the competency and to recognize the necessity of interprofessional cooperation.		
B1.6 The competency "**Students are able to assess CIM methods with regard to their evidence on the basis of corresponding literature research**" is relevant independently of CIM, but here it forms the basis for a critical approach to CIM.		
B1.7 The competency "**Students are able to critically evaluate publications on CIM**" is relevant and should be taught explicitly as a prerequisite for professional action.		
B1.13 The competency "**Students are able to respect different therapeutic perspectives and practices**" is relevant independently of CIM and should be taught explicitly as a prerequisite for interprofessional care.		
B1.17 The competency "**Students are able to advise patients and relatives on CIM in an interprofessional team**" is relevant and serves to identify knowledge gaps in one's own profession on the one hand and to be able to react to the variety of methods and needs used by patients on the other hand.		
B1.14 The competency "**Students are able to inquire and recognize the physical, psychological and social care needs of a patient**" is relevant, but requires the ability to evaluate different options for action.		
B1.4 The competency "**Students are able to expand their knowledge of CIM procedures**" is partly relevant, since students usually have little previous knowledge of the procedures.		
B1.10 The competency "**Students are able to be familiar with the skills of all professions involved in CIM care**" is partly relevant, as it is a prerequisite for appreciative cooperation and communication.		
B1.5 The competency "**Students are able to assess CIM methods with regard to their evidence on the basis of corresponding literature research**" is partly relevant and should be taught rather explicitly. However, not only CIM databases should be used for research.		
B1.18 The competency "**Students are able to handle their own health carefully with awareness**" is partly relevant and should be addressed rather implicitly.		
B1.12 The competency "**Students are able to recognize how their specific occupational roles contribute to provide the necessary patient care when working in interprofessional teams**" is partly relevant and depends on the CIM action possibilities in the respective care situation.		
B1.8 The competency "**Students are able to independently apply selected methods to patient care**" is not relevant. However, independent of this, practical exercises in the module can deepen the learning content and promote critical assessment.		

Others:

Below is a list of the competencies and teaching-learning objectives proposed in the first survey round. You now have the opportunity to assess the relevance on a seven-point Likert scale. Please also indicate whether these competencies should be addressed explicitly or implicitly ("between the lines").

	1=not at all relevant	2	3	4	5	6	7=very relevant	How should the competencies be addressed? rather explicit	rather implicit
B2.1 Students are able to coordinate conventional (orthodox) and complementary medicine as simultaneous care and therapy options.									
B2.2 Students are able to recognize independently extended CIM therapy options in specific care situations.									
B2.3 Students are able to assess how CIM methods fit in with the conventional therapies used in each case.									
B2.4 Students are able to find out the compatibility of and similarities between conventional medicine and CIM.									
B2.5 Students are able to critically assess both conventional medicine and CIM and identify conflicts of interest and commercial structures.									
B2.6 Students are able to point out possibilities for change in the conventional medical care system.									
B2.7 Students are able to know the sociopsychological and historical development of healthcare.									
B2.8 Students are able to use communication techniques respectfully and listen to patients.									
B2.9 Students are able to encounter patients with respect and empathy.									
B2.10 Students are able to take a comprehensive and expanded patient medical history.									

	1=not at all relevant	2	3	4	5	6	7=very relevant	rather explicit	rather implicit
B2.11 Students are able to identify important information based on the patient medical history.									
B2.12 Students are able to make a comprehensive diagnosis based on the information obtained.									
B2.13 Students are able to assess the patient's vegetative reaction and health constitution.									
B2.14 Students are able to recognize the patient's need for advice and information regarding CIM at an early stage and can address this accordingly.									
B2.15 Students are able to give advice on CIM based on an interprofessional multidisciplinary concept.									
B2.16 Students are able to apply counselling strategies that lead to the strengthening of patient autonomy.									
B2.17 Students are able to advise the patient respectfully and competently even if the methods applied by the patient are ineffective, associated with side effects or risks.									
B2.18 Students are able to know the sociopsychological and historical development of healthcare.									
B2.19 Students are able to explain the basics of evidence-based medicine in relation to CIM.									
B2.20 Students are able to distinguish between reliable and non-reliable therapy options.									

	1=not at all relevant	2	3	4	5	6	7=very relevant	rather explicit	rather implicit
B2.21 Students are able to properly evaluate the CIM methods applied.									
B2.22 Students are able to carry out a risk-benefit analysis in relation to CIM procedures.									
B2.23 Students are able to be aware that the application of certain CIM methods and procedures requires its own solid training.									
B1.24 Students are able to recognize different motivations for CIM applications and do not mask patients' fears with CIM therapies.									
B2.25 Students are able to overcome stereotypes and clarify roles in interprofessional communication.									
B2.26 Students are able to discuss and make decisions about CIM in interprofessional communication.									
B2.27 Students are able to develop interprofessional applicable CIM assessments in a team.									
B2.28 Students are able to learn simple CIM methods and thereby extend their scope of action.									
B2.29 Students are able to represent old and new therapy methods in a historical context.									

Here you can justify the assessments you have made and/or list which aspects of the listed competencies you consider particularly important. Please indicate the corresponding number (B2.1-B2.29).

NEXT

2nd Delphi Round: CanMEDS Role Competencies (Part C)

Competencies in medical education are nationally and internationally oriented to the CanMEDS. This competency model was developed for physicians by the Royal College of Physicians and Surgeons of Canada in 2005 and is based on different roles that can be assumed by the respective professions in healthcare.
In the first survey roun, role competencies were assessed on a seven-point Likert scale (1=not at all relevant to 7=very relevant). In the evaluation these were classified into the following four categories:

highly relevant (at least 80% of the respondents rated it with a 6 or 7 on the Likert scale)
relevant (at least 80% of the respondents rated it with a 5-7 on the Likert scale)
partly relevant (at least 80% of the respondents rated it with a 4-7 on the Likert scale)
not relevant (remaining topics)
Furthermore, the free-text fields were evaluated and hypotheses were derived. The hypotheses and the newly listed Role competencies are now presented to you for evaluation.

In the following, competencies are arranged in descending hierarchical order according to the relevance assessment already carried out, but retains its original numbering from the first survey wave. Please indicate whether you agree with the groupings and hypotheses.
If you fully agree with the statement, please tick the respective box on the left side of the page.
If you do not agree or only partially agree with the statement or would like to add something, please use the free text field behind the statement.

	I agree	Free-field box
C1.3 The competency "**Graduates behave in an appreciative manner in interprofessional cooperation and thus contribute to good patient care**" is one of the most important sub-competency for an interprofessional CIM curriculum.		
C1.8 The competency "**Graduates work together with various health professions and disciplines in an appropriate, appreciative and efficient manner**" is also very relevant.		
C1.4 The competency "**Graduates have a professional role identity and know the roles, competencies and responsibilities of the other professional groups involved**" is relevant.		
C1.2 The competency "**Graduates develop concepts, guidelines, treatment pathways, etc. in cooperation with other healthcare professions which can support and further the quality of care**" is relevant.		
C1.1 The competency "**Graduates participate actively and constructively in the teamwork for the joint accomplishment of tasks**" is relevant.		
C1.7 The competency "**Graduates reflect their tasks with regard to continuous patient care**" is relevant.		
C1.5 The competency "**Graduates recognise interprofessional conflicts and actively contribute to productive and appropriate solutions**" is relevant.		
C1.6 The competency "**Graduates explain their role identity (tasks and duties) to other**s" is relevant.		
C1.10 The competency "**Together with the other team members, graduates evaluate the quality of the joint work and, if necessary, agree on measures for improvement**" is only partially relevant.		
C1.9 The competency "**Graduates analyse existing healthcare structures when interacting with other healthcare professions and evaluate them in terms of their effectiveness and efficiency**" is only partially relevant.		

In the first survey round, the other CanMEDs roles were evaluated: Scholar, Communicator, Health Advocate, Leader, Professional and Medical Expert.
The Communicator was rated as very relevant and the Professional, Medical Expert and Scholar as relevant for a CIM curriculum. The sub-competencies of these four role competencies are now presented one after the other for evaluation.
Please indicate which of the listed CanMEDS sub-competences you consider relevant for interprofessional teaching in the context of interprofessional CIM care!

1. Sub-competencies: Communicator

	1=not at all relevant	2	3	4	5	6	7=very relevant
C2.1 Graduates recognise the central importance of communication skills for their profession and healthcare and know that communication can be learned.							
C2.2 Graduates create a trusting, stable doctor/-patient relationship and master a professional and patient-centred approach to conversation, considering the specific types of conversations, phases of conversations and tasks involved.							
C2.3 Graduates reflect on typical sensitive topics in everyday professional life and shape their communication appropriately, even in emotionally challenging situations							
C2.4 Graduates successfully shape their communicative actions through the targeted use of communication strategies, even in challenging clinical contexts and constellations.							
C2.5 Graduates analyse and reflect on sociodemographic and socioeconomic factors influencing communication, and communicate accordingly.							
C2.6 Graduates reflect on the specific requirements of oral, written and electronic communication, as well as of public communication, and interact context-specifically while respecting data protection.							

Here you can justify the assessments made on the CanMEDS sub-competencies of the communicator and/or list which aspects you consider particularly important. Please indicate the corresponding competency number (C2.1-C2.6).

2. Sub-competencies: Professional

	1=not at all relevant	2	3	4	5	6	7=very relevant
C3.1 Graduates orient their actions towards values that are central to their professional activity.							
C3.2 Graduates know and consider the legal framework and obligations as well as the ethical principles of professional conduct.							
C3.3 Graduates know and consider the requirements of good clinical and scientific practice.							
C3.4 Graduates have basic ethical skills and abilities.							
C3.5 Graduates know and consider strategies for error and risk management.							
C3.6 Graduates know and consider the needs of people with disabilities.							
C3.7 Graduates know and consider the characteristics, tasks, responsibilities and development of the profession.							
C3.8 Graduates are familiar with the basics of the economic framework and health policy.							
C3.9 Graduates contribute to quality assurance.							
C3.10 Graduates consider their own health and well-being as a prerequisite for a professional career.							
C3.11 Graduates are capable of self-knowledge, self-reflection, self-criticism and self-development.							
C3.12 Graduates deal with various forms of uncertainty.							
C3.13 Graduates know the prerequisites and measures to establish trust.							
C3.14 Graduates are able to deal with role-related challenges.							
C3.15 Graduates consider cultural, contextual and everyday aspects in their actions.							

Here you can justify the assessments made on the CanMEDS sub-competencies of the professional and/or list which aspects you consider particularly important. Please indicate the corresponding competency number (C3.1-C3.15).

3. Sub-competencies: Medical Expert

	1=not at all relevant	2	3	4	5	6	7=very relevant
C4.1 Graduates carry out an ethically founded and patient-centred medical care appropriate to their educational level, integrating all professional roles.							
C4.2 Graduates apply their knowledge, skills and professional behaviour (attitudes) and keep them up to date.							
C4.3 Graduates carry out an appropriate diagnosis of their patients.							
C4.4 Graduates use preventive, therapeutic and rehabilitative measures effectively.							

Here you can justify the assessments made on the CanMEDS sub-competencies of the Medical Expert and/or list which aspects you consider particularly important. Please indicate the corresponding competency number (C4.1-4.4).

4. Sub-competencies: Scholar

	1=not at all relevant	2	3	4	5	6	7=very relevant
C5.1 Graduates master principles of learning in terms of recognising, reflecting on and meeting one's own learning needs and implementing learning outcomes.							
C5.2 Graduates can design and document an adequate learning process based on a learning question.							
C5.3 Graduates apply the principles of critical evaluation of scientific sources.							
C5.4 Graduates collect and critically evaluate primary and secondary evidence on a medical question.							
C5.5 Graduates integrate conclusions based on critical evaluation into their daily work.							
C5.6 Graduates recognise the principles of learning in their relevance to training in medicine and other healthcare professions and apply them.							
C5.7 Graduates reflect on and evaluate teaching/learning situations.							
C5.8 Graduates internalise the principles of research and scientific questioning.							
C5.9 Graduates know the ethical and legal principles of research.							
C5.10 Graduates contribute to the development of new knowledge.							

Here you can justify the assessments made on the CanMEDS sub-competencies of the Scholar and/or list which aspects you consider particularly important. Please indicate the corresponding competency number (C5.1-C5.10).

NEXT

2nd Delphi Round: Curricular Content (Part D)

In the first survey round, general curricular content were assessed on a seven-point Likert scale (1=not at all relevant to 7=very relevant). In the evaluation these were classified into the following four categories:
highly relevant (at least 80% of the respondents rated it with a 6 or 7 on the Likert scale)
relevant (at least 80% of the respondents rated it with a 5-7 on the Likert scale)
partly relevant (at least 80% of the respondents rated it with a 4-7 on the Likert scale)
not relevant (remaining topics)

Furthermore, the free-text fields were evaluated and hypotheses were derived. The hypotheses and the newly listed content are now presented to you for evaluation.

In the following, general curricular content is arranged in descending hierarchical order according to the relevance assessment already carried out, but retains its original numbering from the first survey wave. Please indicate whether you agree with the groupings and hypotheses.
If you fully agree with the statement, please tick the respective box on the left side of the page.
If you do not agree or only partially agree with the statement or would like to add something, please use the free text field behind the

	I agree	Free-text field
D1.9 The content **"Overview of methods in classical natural medicine"** is relevant.		
D1.10 The content **"Overview of methods in non-classical natural medicine"** is relevant.		
D1.2 The content **"Clarification of CIM-Terms: Naturopathy, Complementary Medicine, Alternative Medicine, Integrative Medicine, etc."** is relevant in order to find a common language and prevent misunderstandings.		
D1.8 The content **"Overview of effects and interactions of selected CIM therapies"** is relevant.		
D1.5 The content **"Supply structures and service providers"** is partly relevant, since explicit knowledge, for example on the qualification of service providers, increases patient safety.		
D1.3 The content **"Legal issues relating to CIM therapies regarding delegation, substitution and reserved activities within CIM patient care"** is partly relevant and should be based on an overview.		
D1.7 The content **"Deepening the assessment of CIM evidence on the basis of selected studies"** is partly relevant, in particular because it allows the individual to critically reflect on his own actions.		
D1.6 The content **"Overview of CIM databases and literature search via the Internet"** is partly relevant, but all relevant databases should be included in the literature search.		
D1.4 The content **"Use of CIM therapies"** is partly relevant.		
D1.1 The content **"Historical and philosophical backgrounds of CIM therapy"** is not relevant, but can be discussed if it is important as a basis for understanding the therapy method.		
Others:		

The following general curricular content was additionally suggested in the first survey round. Please rate it on a seven-point Likert scale. You also have the option to indicate if you cannot or do not want to assess the content due to lack of knowledge and/or other reasons.

	1=not at all relevant	2	3	4	5	6	7=very relevant	I feel not competent to assess
D2.1 Placebo (effect and possibilities)								
D2.2 Motives and reasons for using CIM therapies								
D2.3 Health of health professionals, resilience and burn-out prevention								
D2.4 Obstacles to and opportunities for using CIM from a provider perspective								
D2.5 Motivation for the demand for CIM services in the population								
D2.6 CIM in guidelines: Information and critical reflection								

Here you can justify the assessments you have made and/or list which aspects of the listed content you consider particularly important. Please indicate the corresponding number (D.2.1-D.2.6).

Regardless of how the module is structured, the content should be deepened on the basis of concrete treatment reasons. In your opinion, which treatment reasons are particularly suitable to illustrate the indication, application and effects of CIM?
Already mentioned:
Infectious diseases like urinary tract infections and bronchitis
Susceptibility to infections
Arterial hypertension
Back Pain
Climacteric complaints
Complaints caused by chemotherapy

Here you have the possibility to list further treatment reasons.

Classical natural medicine

The five tenets of classical natural medicine (hydrotherapy, balance of mind and body, exercise, nutrition and herbalism) were assessed as relevant in the first survey round. In the following, these "tenets" are now broken down into individual procedures. The procedures were assigned to the tenets, whereby it should be noted that there are also overlaps in many areas.
Please rate the relevance of the respective procedures on a seven-point Likert scale. In addition, you have the option to indicate if you cannot or do not want to assess the content due to lack of knowledge and/or other reasons.

	1=not at all relevant	2	3	4	5	6	7=very relevant	I feel not competent to assess
D3.1 Hydrptherapy: Kneipp therapy (Bath, washings, pourings)								
D3.2 Hydrptherapy: Wraps and compresses								
D3.3 Regulative therapy / Mind-Body medicine: Yoga								
D3.4 Regulative therapy / Mind-Body medicine: Progressive muscle relaxation								
D3.5 Regulative therapy / Mind-Body medicine: Art therapy								
D3.6 Regulative therapy / Mind-Body medicine: Stress management								

D3.7 Regulative therapy / Mind-Body medicine: Mindfulness and meditation								
D3.8 Regulative therapy / Mind-Body medicine: Digital forms of relaxation (My headspace for sleep therapy)								
D3.9 Movement therapy: Exercise and endurance training								
D3.10 Movement therapy: Dance and music therapy								
D3.11 Movement therapy: Fascia therapy								
D3.12 Dietetics: General nutritional recommendations (Nutrient supply, vitamins and trace elements)								
D3.13 Dietetics: Diet for special wishes (Mediterranean, vegan, low-carb, ...)								
D3.14 Dietetics: Nutrition for food intolerances and allergies								
D3.15 Dietetics: High-dose vitamins (orthomolecular therapy)								
D3.16 Dietetics: Fasting (healing fasting, interval fasting								
D3.17 Phytotherapy: Medicinal plants, tea, tinctures								
D3.18 Phytotherapy: Aromatherapy, embrocation								

Here you can justify the assessments you have made and/or list which aspects of the listed content you consider particularly important. Please indicate the corresponding number (D.3.1-D.3.18).

In the survey sound, the non-classical natural medicine methods were classified as partly relevant or not relevant, although in some cases the exemplary integration was desired. Please indicate below to what extent you agree with the statements made here. Click on the left-hand box if you fully agree with the statement. In the right box you can write a comment if you only partially agree with the statement or if you have further comments.

Non-classical natural medicine is understood here to be all those traditional procedures that have either been well researched or proven by experiential healing knowledge.

Clearly distinguishable from this are paramedical procedures. These do not have a corresponding tradition and thus no correspondingly extensive experience.

	1=not at all relevant	2	3	4	5	6	7=very relevant	I feel not competent to assess
D4.1 As many non-classical naturopathic treatments as possible should be discussed so that the students get a good overview.								
D4.2 Non-classical naturopathic therapies for which a scientifically proven efficacy is available should be the main topic.								
D4.3 The non-classical naturopathic therapies often desired ba patients should primarily be discussed.								
D4.4 The non-classical naturopathic therapies that are frequently offered locally and discussed in public should be addressed primarily.								
D4.5 When discussing non-classical naturopathic treatments, the topic of acupuncture must be covered.								
D4.6 When discussing non-classical naturopathic treatments, the choice of lecturers should be made carefully in order to enable an objective and scientific approach.								
D4.7 Paramedical procedures should also be addressed in order to sensitise students to shalatanery.								

Non-classical natural medicine

The following non-classical natural healing methods were suggested in the first survey round as possible additional content (in addition to acupuncture, homeopathy, Ayurveda, etc.). In the following, these procedures are now broken down.

	1=not at all relevant	2	3	4	5	6	7=very relevant	I feel not competent to assess
D5.1 Osteopathy								
D5.2 Classical massages								
D5.3 Shiatsu (Manual therapy method, application of pressure and friction to certain areas of skin)								
D5.4 Tai Chi (origin in the martial arts, today rather meditatively performed)								

Here you can justify the assessments you have made and/or list which aspects of the listed content you consider particularly important. Please indicate the corresponding number (D.5.1-D.5.4).

Paramedical procedures

The following paramedical procedures were suggested as possible content in the first survey round. These procedures are now broken down in the following.

	1=not at all relevant	2	3	4	5	6	7=very relevant	I feel not competent to assess
D6.1 (Foot zone) reflex therapy								
D6.2 Hildegard medicine								
D6.3 Iris diagnostics								
D6.4 Bach flowers								
D6.5 Schuessler salts (homeopathically administered mineral salts)								
D6.6 Kinesiology								
D6.7 Reiki (Energy work, technique of laying on of hands)								
D6.8 Color therapy								

Here you can justify the assessments you have made and/or list which aspects of the listed content you consider particularly important. Please indicate the corresponding number (D.6.1-D.6.8).

You have reached the end of the interview. Is there anything else you'd like to share?

SUBMIT

Delphi Study: Curriculum Development for Interprofessional Education on Complementary and Integrative Medicine

Third Round

We welcome you to the third round of the Delphi survey "Development of an interprofessional curriculum on complementary and integrative medicine"

Thank you very much for participating in the first and second survey rounds!

This third survey round is somewhat shorter and will take up a few open questions. It is very important that you work through this survey completely, because at the end you will have the opportunity to provide some personal information on how you would like to be further involved.

The survey is divided into four short parts: Part A: General questions, B: Questions about treatment reasons and disease patterns, Part C: Questions about role competencies, Part D: Personal data and feedback.

It should take about 10 to 20 minutes to complete the questionnaire.

You can stop and come back to continue the questionnaire at a later time. Your results will be stored temporarily.

If you have any questions, please do not hesitate to contact me.

Angelika Homberg
Phone +49 (0) 6221 567169
angelika.homberg@med.uni-heidelberg.de

NEXT

3rd Delphi Round: General Questions (Part A)

In the following, you will be presented with some questions that pick up critical discussion points from the first two survey rounds.
When answering the questions, please refer to a potential seminar for Complementary and Integrative Medicine (CIM) with 30-60 teaching units of 45 min each, which is offered at a medical school for students of medicine and other healthcare professions (from the fields of nursing, therapy and diagnostics) in undergraduate education.

In your opinion, what are the greatest barriers and/or challenges when offering an interprofessional CIM seminar at a medical school?	
In your opinion, what are the greatest benefits and/or opportunities when offering an interprofessional CIM seminar at a medical school?	

In the first two survey rounds it was noted, among other things, that it may be difficult to recruit lecturers with the appropriate technical and professional background for teaching at CIM. It would, however, be possible to offer individual CIM topics as e-learning units.
Please indicate to what extent you agree with the following statements!

	I completely agree	I rather agree	I partly agree	I rather disagree	I disagree at all
E-learning units are particularly suitable for presenting theoretical background information on CIM concepts and therapy methods.					
E-learning units are particularly suitable for presenting case-based scenarios for individual treatment causes in a CIM seminar.					
E-learning units are particularly suitable for presenting different professional perspectives on a CIM topic.					
E-learning units should not be integrated in an interprofessional CIM seminar.					
When integrating e-learning units into a CIM seminar, care must be taken to ensure that there is sufficient attendance time available for the exchange between students.					
An interprofessional CIM seminar could be completely covered by e-learning units.					

What other barriers and benefits do you see in offering e-learning units in an interprofessional CIM seminar?	
Here you have the opportunity to comment or supplement your statement on e-learning units in a CIM seminar.	

The integration of practical exercises of CIM procedures and methods was discussed in the first two rounds of the survey and several times requested. On the other hand, it was noted that this only makes sense if they are also used later in everyday professional life.

Please comment on the following statements!

	I completely agree	I rather agree	I partly agree	I rather disagree	I disagree at all
Hands-on experience should be integrated into a CIM seminar so that students can experience and assess the effect of the methods themselves.					
Hands-on experience should be integrated into a CIM seminar so that students can assess the feasibility/workability of the respective method.					
Hands-on experience should be integrated into a CIM seminar so that students can better understand and comprehend the respective therapeutic concept.					
Hands-on experience should be integrated into a CIM seminar so that students acquire the ability to apply the methods in professional practice.					
Hands-on experience should not be integrated into a CIM seminar.					

Here you have the opportunity to comment or supplement your statement on the acquisition of communicative competencies in a CIM seminar.	
Please name in the following simple therapy methods which you think are particularly suitable to be taken up in a CIM curriculum.	

On the one hand, the promotion of communicative competencies was seen as central to an interprofessional CIM seminar, on the other hand, the risk was mentioned that, if a focus is set in this area, the contents will become too blurred with other contents in the curriculum.
Please comment on the following statements!

	I completely agree	I rather agree	I partly agree	I rather disagree	I disagree at all
The acquisition of communicative skills should be the main focus of an interprofessional CIM seminar from the very beginning.					
Students should first be given an overview of CIM before the acquisition of communicative competencies is given priority.					
The acquisition of communicative skills should not be given priority in a CIM seminar, as otherwise there is a risk that other content will be neglected.					

Here you have the opportunity to comment on your statement on communicative competencies.	

NEXT

3rd Delphi Round: Treatment Reasons and Disease Patterns (Part B)

In tis survey round, it was suggested that curricular content should be oriented towards different treatment reasons and/or disease patterns. Case studies on specific treatment reasons or diseases can also facilitate the transfer into professional practice when addressing individual treatment concepts. The following treatment reasons and disease patterns were suggested in the first and second survey rounds.

The following reasons for seeking care were suggested in the first and second survey rounds. The treatment reasons can serve as a basis for developing CIM advice, diagnostics and therapy in an exemplary and practical manner within the seminar. In the following, the terms used to identify reasons for seeking care are based on the National Competence-oriented Catalogue of Learning Objectives in Medicine (NKLM, Chapter 20). The topics are arranged alphabetically.

	1=not at all relevant	2	3	4	5	6	7=very relevant	I feel not competent to assess
B1.1 Abnormal menstrual periods and cycle irregularities								
B1.2 Fear and anxiety								
B1.3 Fever								
B1.4 Generalized pain, pain at multiple localizations								
B1.5 Sore throat								
B1.6 Cough								
B1.7 Headache								
B1.8 Fatigue, exhaustion, general weakness								
B1.9 High-risk pregnancy and pregnancy problems like hyperemesis (vomiting)								
B1.10 Back pain								
B1.11 Insomnia								
B1.12 Pain in the extremities and joints								
B1.13 Pregnancy (without known symptoms)								
B1.14 Complaints without identifiable physical cause								

Here you can justify the assessments you have made and/or list which aspects of the listed content you consider particularly important. Please indicate the corresponding number (B.1.1-B.1.14).

The following disease patterns were suggested in the first and second survey rounds. In the following, the respective nomenclature is based on the National Competence-oriented Catalogue of Learning Objectives in Medicine (NKLM, Chapter 21). The disease patterns are arranged thematically and alphabetically.

	1=not at all relevant	2	3	4	5	6	7=very relevant	I feel not competent to assess
B2.1 Essential hypertension								
B2.2 Obesity								
B2.3 Arthrosis								
B2.4 Arthritis (inflammation of the joints)								
B2.5 Fibromyalgia syndrome								
B2.6 Chronic back pain								
B2.7 Diabetes mellitus type 1								
B2.8 Diabetes mellitus type 2								
B2.9 Metabolic syndrome								
B2.10 Chronic and acute bronchitis								
B2.11 Bronchial asthma								
B2.12 Allergic rhino conjunctivitis/allergic rhinosinusitis (hay fever)								
B2.13 Infections of the kidney and urinary tract (e.g. cystitis)								
B2.14 Menopause, climacteric (menopause)								
B2.15 Chronic inflammatory intestinal diseases								
B2.16 Irritable bowel syndrome								
B2.17 Atopic eczema/neurodermatitis								
B2.18 Migraine								
B2.19 Depression								
B2.20 Anxiety disorders								
B2.21 Burnout								

Here you can justify the assessments you have made and/or list which aspects of the listed content you consider particularly important. Please indicate the corresponding number (B.2.1-B.2.21).

Below is a list of treatment and counseling needs of specific patient groups proposed in the first and second survey rounds. The answers are arranged according to the respective patient groups.

	1=not at all relevant	2	3	4	5	6	7=very relevant	I feel not competent to assess
B3.1 Healthy: Support for the development of self- and health competence								
B3.2 Healthy: Support for susceptibility to infection								
B3.3 Pediatrics: Pediatric diseases								
B3.4 Puerperium: Support for regression								
B3.5 Puerperium: Support during breastfeeding, milk congestion								
B3.6 Injuries and wounds: Support of the healing process								
B3.7 Oncological patients: Support after surgery								
B3.8 Oncological patients: Support during radiation								
B3.9 Oncological patients: Support for complaints caused by chemotherapy								
B3.10 Oncology, survivors: Support for cancer-related fatigue								

Here you can justify the assessments you have made and/or list which aspects of the listed content you consider particularly important. Please indicate the corresponding number (B.3.1.1-B.3.10).

NEXT

3rd Delphi Round: CanMEDS Role competencies (Part C)

Competencies in medical education are nationally and internationally oriented to the CanMEDS. This competency model was developed for physicians by the Royal College of Physicians and Surgeons of Canada in 2005 and is based on different roles that can be assumed by the respective professions in healthcare.

Each of the seven roles is subdivided into a different number of sub-competencies.

In the first survey round, the relevance of the sub-competencies of the CanMEDS role: Collaborator was assessed, in the second round the roles of Scholar, Communicator, Professional and Medical Expert were assessed.

Finally, the sub-competencies of Health Advocate and Leader should now be assessed.

Please indicate which of the listed CanMEDS sub-competencies you consider relevant in the context of interprofessional teaching on CIM!

1. Sub-competencies: Health Advocate

	1=not at all relevant	2	3	4	5	6	7=very relevant
C1.1 Graduates recognise the state of health of individual persons as a whole, as well as imbalances and disparities in the state of health, their causes and consequences.							
C1.2 Graduates work towards reducing imbalances and disparities in the health status of individuals.							
C1.3 Graduates pursue individual health promotion in cooperation with other health professions and service providers.							
C1.4 Graduates recognise the state of health of patients and population groups, as well as imbalances and disparities in the state of health, their causes and consequences.							
C1.5 Graduates work towards reducing imbalances and disparities in the health status of patients and population groups.							
C1.6 Graduates pursue individual and population-related health promotion in cooperation with institutions and organisations in the healthcare system.							
C1.7 Graduates conduct population-based health promotion in cooperation with other health professionals and service providers.							

Here you can justify the assessments made on the CanMEDS sub-competencies of the communicator and/or list which aspects you consider particularly important. Please indicate the corresponding competency number (C1.1-C1.7).

2. Sub-competencies: Leader

	1=not at all relevant	2	3	4	5	6	7=very relevant
C2.1 Graduates reflect on their own role as a responsible person and manager in the health system.							
C2.2 Graduates identify and analyse social care structures.							
C2.3 Graduates describe the basic health economic structures and interrelationships and are able to apply this knowledge in the concrete context of patient care and participate in problem solving.							
C2.4 Graduates are proficient in effective and efficient medical work, show and define problems and develop approaches to solutions.							
C2.5 Graduates recognise situations in patient care in which decisions have to be made on the distribution of resources and participate in the decision-making process.							
C2.6 Graduates know the methods of resource allocation (at different levels and for different service providers).							
C2.7 Graduates have knowledge of measures for quality assurance in patient care and their areas of application.							
C2.8 Graduates address essential aspects of dealing with errors and use strategies for implementing patient safety.							
C2.9 Graduates know essential aspects of complication management, risk communication, CIRS (Critical Incident Reporting System), recognise critical events and are trained in dealing with wrong decisions.							
C2.10 Graduates demonstrate an appropriate handling of adverse events and failures.							
C2.11 Graduates use information technology (IT) to obtain and transfer information and to document treatment processes.							
C2.12 Graduates develop strategies for setting priorities and effective time management.							
C2.13 Graduates deal with the role of a young team member and their future development as a leader.							
C2.14 Graduates plan their own career and identify their personal qualification needs.							
C2.15 Graduates develop professional goals at the beginning of job and career planning and consider and communicate the work/life balance when implementing these goals.							
C2.16 Graduates know management styles and take on management tasks.							

Here you can justify the assessments made on the CanMEDS sub-competencies of the communicator and/or list which aspects you consider particularly important. Please indicate the corresponding competency number (C2.1-C2.16).

NEXT

3rd Delphi Round: Personal Data, Feedback (Part D)

Finally, you have the opportunity to indicate whether and in what form you would like to be involved in the publication of the results and further development of the curriculum. Furthermore, you have the opportunity to evaluate the survey process.

The data collected in the three survey waves will be evaluated and published by the working group of General Medicine and Health Services Research at the University of Heidelberg. Please indicate whether and in what form you would like to participate, be named and/or informed!

	Yes	No	No answer
I would like to be informed about corresponding publications.			
I am willing to actively participate in publications as co-author.			
My name (first and last name) may be mentioned in the acknowledgements of publications. However, no connections may be made between individual statements or results concerning my person or corresponding conclusions may be drawn.			

Here you can add further comments on the topic "Publications" and/or describe in which form you can or want to get involved!

A curriculum consists not only of competencies and content, but also of a coherent concept that also integrates teaching methods. The development of such a concrete curriculum goes beyond the scope of a written survey. However, the results of this survey can be used as a basis for the development of a concrete curriculum.
[illegible]
work on the further development of the curriculum. Travel and accommodation costs will be covered, but a fee cannot be paid.

	Yes	No	No answer
I am willing to take part in the workshop (one day)			
Independent of the participation/non-participation in the mentioned workshop, I will gladly be available for further questions and discussions regarding the further development of an interprofessional CIM curriculum.			

I have the following remarks, suggestions and wishes for the workshop or for further cooperation.

With the following two questions at the end of the survey I would like to find out how you generally got along with the Delphi survey.

At this point you can name difficulties and improvement wishes as well as perceived bias of the surey process!

At this point you can name successful and positive aspects of the survey process!

Thank you very much for completing the questionnaire! Is there anything else you would like to share?

MANY THANKS FOR YOUR COMMITMENT!

SUBMIT
